# Prepubertal Growth Trajectory and Pubertal Onset

**DOI:** 10.1001/jamanetworkopen.2026.17435

**Published:** 2026-06-09

**Authors:** Rui Deng, Weiqin Li, Jiali Duan, Tianshu Feng, Liumei Wei, Xingxiu Li, Junhong Leng, Xijie Wang, Bin Dong, Susan M. Sawyer

**Affiliations:** 1 Institute of Nutrition and Food Hygiene, Beijing Center for Disease Prevention and Control, Beijing, China; 2 Tianjin Women and Children’s Health Centre, Tianjin, China; 3 Institute of Child and Adolescent Health, School of Public Health, Peking University Health Science Centre, Beijing, China; 4 School of Disaster and Emergency Medicine, Tianjin University, Tianjin, China; 5 Beijing Key Laboratory of Innovations and Transformations in Intelligent and Precise Diagnosis and Treatment Technologies for Reproductive Health, Beijing, China; 6 Centre for Adolescent Health, Murdoch Children’s Research Institute and Royal Children’s Hospital, Parkville, Victoria, Australia; 7 Department of Paediatrics, The University of Melbourne, Parkville, Victoria, Australia

## Abstract

**Importance:**

Adiposity has been reported to be a major contributor to earlier pubertal timing, but most pediatric studies have relied on body mass index (BMI) from single or short-term measurements. Studies tracking growth repeatedly from birth to puberty are needed.

**Objective:**

To explore the association of growth trajectory and cumulative exposure to different levels of adiposity (CEA) with pubertal onset across the first decade of life and identify sensitive periods for possible weight intervention.

**Design, Setting, and Participants:**

This population-based cohort study included data from 2 birth cohorts: the Longitudinal Study of Australian Children (LSAC), conducted from March 2004 to September 2021, with more than a 10-year follow-up, and the Tianjin Birth Cohort Study (TBCS) in China, conducted from May 2021 to April 2024, with follow-up from December 2010 to April 2024. Data analysis was conducted from April 2023 to November 2025. Participant inclusion required at least 4 anthropometric measures in the LSAC and 9 in the TBCS and completed measures of pubertal onset in both cohorts.

**Exposures:**

Prepubertal BMI trajectories, CEA, and rates of BMI increase at each age.

**Main Outcomes and Measures:**

Pubertal status and timing were obtained by a parent-reported Pubertal Development Scale. A latent class growth mixed model was used to identify prepubertal BMI trajectories. An interval regression model and the Cox proportional hazards regression model were used to examine the association of the exposures with the age and risk of pubertal onset.

**Results:**

A total of 3354 Australian children (1723 boys [51.37%]) and 1105 Chinese children (563 girls [50.95%]) were included. At the last round, the mean (SD) ages were similar across sexes within each cohort, while children in LSAC (14.83 [0.61] years) were overall older than those in TBCS (10.63 [0.60] years). Girls in BMI trajectory groups characterized as high-level or increasing were younger at pubertal onset (from β = −0.36 [95% CI, −0.66 to −0.07] years to β = −1.51 [95% CI, −2.68 to −0.35] years) and were associated with increased risk of pubertal initiation (from hazard ratio [HR], 1.35 [95% CI, 1.04 to 1.74] to HR, 2.80 [95% CI, 1.69 to 4.63]). A higher CEA and average CEA (both >2) were associated with an earlier age at pubertal onset (from β = −0.04 [95% CI, −0.05 to −0.03] years to β = −0.85 [95% CI, −1.48 to −0.23] years), with a greater effect size after averaging. Consistent results were found in boys of the LSAC but not those of the TBCS. Sensitive ages of 3 to 4 years were identified, at which BMI increase was associated with pubertal timing, with greater effect sizes of pubertal timing (from β = −1.35 [95% CI, −2.00 to −0.71] years to β = −3.41 [95% CI, −4.13 to −2.68] years) and greater risk of pubertal onset (from HR, 1.85 [95% CI, 1.29 to 2.67] to HR, 5.59 [95% CI, 3.73 to 8.37]) than at other ages. Notably, the effect sizes of CEA within this period were greater than that outside it.

**Conclusions and Relevance:**

In this cohort study, high-level or increasing prepubertal growth trajectories and greater CEA were associated with earlier and higher risk of pubertal onset. These findings highlight the importance of considering CEA in relation to early pubertal onset and suggest that ages 3 to 4 years may be an important intervention period for earlier pubertal onset monitoring.

## Key Points

**Question:**

Is cumulative exposure to adiposity before puberty associated with pubertal onset?

**Findings:**

In this cohort study that included 2 birth cohorts of 4459 children, high-level or increasing prepubertal growth trajectories and greater cumulative exposure to adiposity were associated with earlier and higher risk of pubertal onset. Ages 3 to 4 years were identified as the potential sensitive period for rapid growth associated with early pubertal timing.

**Meaning:**

These findings suggest that children with greater cumulative exposure to adiposity before puberty may need to be monitored for earlier pubertal onset, with particular attention to the critical window of ages 3 to 4 years.

## Teaser

This cohort study explores the association of prepubertal growth trajectory and cumulative exposure to adiposity with pubertal onset and evaluates potential sensitive periods for preventive intervention.

## Introduction

Secular trends around the timing of puberty have been apparent for at least the past 100 years.^1,2,3^ Although a recent study in a largely Australian cohort suggests that the age of menarche may have stabilized,^4^ there is little evidence to support that the age of onset of other pubertal development events has also stabilized. Emerging research shows a parallel rise in the prevalence of early puberty onset since the COVID-19 pandemic.^5,6^ Early initiation of puberty is associated with increased risk of behavioral and emotional problems during adolescence and with noncommunicable diseases in adulthood, including cardiac disease and gynecologic cancers.^7,8,9^ In this context, greater understanding of the factors influencing pubertal onset is indicated.

Evidence suggests that higher body mass index (BMI) is associated with earlier pubertal onset.^10,11^ However, most pediatric studies have relied on BMI from single or short-term prepubertal measurements, with few tracking growth repeatedly from birth to puberty.^12,13,14^ Even among these few studies, most have not accounted for the persistence of different adiposity levels.^15,16^ Cumulative exposure to adiposity, accounting for both the duration of obesity among individuals and the severity of their adiposity,^17^ has been shown to be a better predictor of diabetes risk in adults than the duration of adiposity or the magnitude of adiposity alone.^18^ Studies have also shown that cumulative exposure to adiposity from adolescence to adulthood is relevant for adult cardiovascular disease risk factors.^19^ Among children, it remains unknown how cumulative exposure to adiposity from birth may affect pubertal timing.

Beyond absolute BMI, a rapid BMI increase may be related to earlier pubertal onset. Most previous studies used changes of BMI values across a limited period of time to evaluate children’s growth.^12,20,21,22^ In the absence of studies that span from birth across the first decade, it is still unclear whether there is a specific window within which rapid weight gain has the strongest effect on earlier pubertal onset. While adult studies have used BMI slopes to objectively quantify growth velocity, no pediatric study, to our knowledge, has applied this method to identify a sensitive period for BMI increase associated with pubertal timing.^23,24^

To address these gaps, we leveraged data from 2 birth cohorts from Australia and China and set out to characterize BMI trajectories from birth across the first decade, examine the associations of cumulative exposure to different levels of adiposity (CEA) with pubertal timing, and identify potential sensitive periods for preventive intervention.

## Methods

### Data Sources

This cohort study included data from 2 birth cohorts. The Longitudinal Study of Australian Children (LSAC) is a nationally representative birth cohort of children’s development and well-being in Australia. Detailed information on the study sampling and technical design has been reported.^25^ We used the LSAC study’s B cohort, which recruited Australian-born children between 2003 and 2004. Children were followed from wave 1 (baseline, at birth) to wave 8 (end point, ages 14-15 years). Our study dates were from March 2004 to September 2021, with more than a 10-year follow-up. Anthropometric measurements were conducted every 2 years from birth. Pubertal status was assessed biennially from wave 5 in girls and wave 6 in boys. All children with an anthropometric measurement and pubertal assessment were initially eligible. In our final analyses, the requirement for complete data (ie, the minimum amount of anthropometric measurements and pubertal assessments) was presented as an inclusion criterion for children included in the study (eFigure 1 and eTable 1 in Supplement 1). Ethical approval for the LSAC was obtained from the Australian Institute of Family Studies Ethics Committee. Parents or guardians provided written informed consent. This study followed the Strengthening the Reporting of Observational Studies in Epidemiology (STROBE) reporting guideline for cohort studies.

The Tianjin Birth Cohort Study (TBCS) consists of children selected from 3 elementary schools in the Xiqing District, Tianjin, China. Study dates were from May 2021 to April 2024. Anthropometric measurements from birth to age 6 years were obtained from medical records from the Tianjin Women and Children’s Health Centre (12 waves at specified ages). From ages 7 to 10 years, anthropometric measurements data were collected in subsequent follow-up surveys (3 waves). These follow-up surveys also collected detailed information about the children, with semiannual pubertal assessments obtained by parent-reported questionnaires. Based on the same initially eligible criterion and further analytic inclusion criterion, children were included in this study (eFigure 1 and eTable 2 in Supplement 1), from wave 1 (baseline, at birth) to wave 15 (end point, ages 10 to 11 years). Ethical approval was provided by the Ethics Committee of Peking University, and all participants and their parents provided written informed consent.

### Measurements

#### BMI

BMI measurements are detailed in the eMethods in Supplement 1. In both studies, BMI at each wave was calculated as weight in kilograms divided by height in meters squared and then converted into age-specific and sex-specific BMI *z* scores based on the World Health Organization growth references.

#### Pubertal Status

The parent-reported Pubertal Development Scale, developed and validated by Petersen et al,^26^ consisting of 5 items, in which total scores indicate whether puberty was initiated, was used to assess pubertal status in both cohorts (eMethods in Supplement 1). Evidence has indicated that the scale’s scores capture basal hormones, both gonadal and adrenal hormones, and align with physical examinations.^27^ The parent-reported Pubertal Development Scale demonstrated good internal consistency and high test–retest reliability.^28^

### Statistical Analysis

#### BMI Trajectory Groups

We used a latent class growth mixed model to identify different trajectory patterns of BMI *z* scores for each sex. The trajectories were calculated before the age at the last round for prepubertal children or the age at the first report of having initiated puberty for pubertal children. The best-fitting models were cubic trajectories with 5 groups for the LSAC and 4 groups for the TBCS. Details of the model-fitting process were provided in the eMethods and eTables 3 to 10 in Supplement 1.

#### CEA

Based on the model parameters estimated by a latent class growth mixed model, individual curve parameters for each child were then generated (eMethods in Supplement 1). With the World Health Organization classifying a BMI *z* score of 1 or less SDs as normal weight or underweight, BMI *z* scores above 1 SD correspond to varying gradations of adiposity. We thus used the composite trapezoid rule to derive the area under the curve to represent CEA above 1 SD, including cumulative exposure to BMI *z* scores within the range of more than 1 to 2 or less (CE1) and cumulative exposure to BMI *z* scores above 2 (CE2) (eFigure 2 in Supplement 1).^19^ Corresponding exposure durations were calculated similarly. The average cumulative exposure (ACE) to BMI *z* scores within the range of more than 1 to 2 or less (ACE1) and above 2 (ACE2) were then obtained through dividing the cumulative exposure by the corresponding duration. These indicators were all calculated before the age at the last round for prepubertal children or the age at the first report of having initiated puberty for pubertal children. To evaluate a child’s BMI status across the whole follow-up period, another categorical variable of overall BMI status was derived according to whether the BMI *z* scores ever exceeded 1 or 2.

#### Rate of Increase of BMI *z* Scores

Based on the individual curves, linear slopes were derived by calculating the first derivatives at each age in half-year intervals. This represented the rate of increase of BMI *z* scores,^23,24^ either before the age at the first report of having initiated puberty for pubertal children or the age at the last round for prepubertal children.

#### Association Analysis

Data analysis was conducted from April 2023 to November 2025. This study conducted association analyses based on 2 types of pubertal onset outcomes: the age at pubertal onset (continuous variable) and the occurrence of pubertal onset by the last round (categorical variable), to examine their associations with prepubertal BMI trajectories, CEA, and rates of BMI increase at each age. For age at pubertal onset, interval regression models based on the normal distribution were applied, and results were reported as β with 95% CIs. For the occurrence of pubertal onset, Cox proportional hazards regression models and discrete-time survival models were used, and hazard ratios (HRs) with 95% CIs were reported. Detailed information along with the nonlinear association is described in the eMethods and eTable 11 in Supplement 1.

The estimation of latent class growth mixed models was performed with the lcmm package in R, version 4.2.2 (R Project for Statistical Computing). Other analyses were conducted in Stata, version 16.0 (StataCorp LLC), with a 2-sided *P* < .05 considered statistically significant.

## Results

### Basic Characteristics

Among the LSAC study’s cohort of 5107 children, a total of 3354 Australian children (1631 girls [48.63%] and 1723 boys [51.37%]) were included, and of the TBCS’s total cohort of 1510 children, 1105 Chinese children (563 girls [50.95%] and 542 boys [49.05%]) were included. At the last round, the mean (SD) ages were similar across sexes within each cohort, while children in LSAC (14.83 [0.61] years) were overall older than those in TBCS (10.63 [0.60] years). By the last round, in both cohorts, the proportion of pubertal onset in girls (LSAC: 1609 [98.65%]; TBCS: 456 [80.99%]) was significantly higher than that in boys (LSAC: 1575 [91.41%]; TBCS: 319 [58.86%]) (eTable 12 in Supplement 1).

### Trajectories of BMI *z* Scores

**Figure 1.**
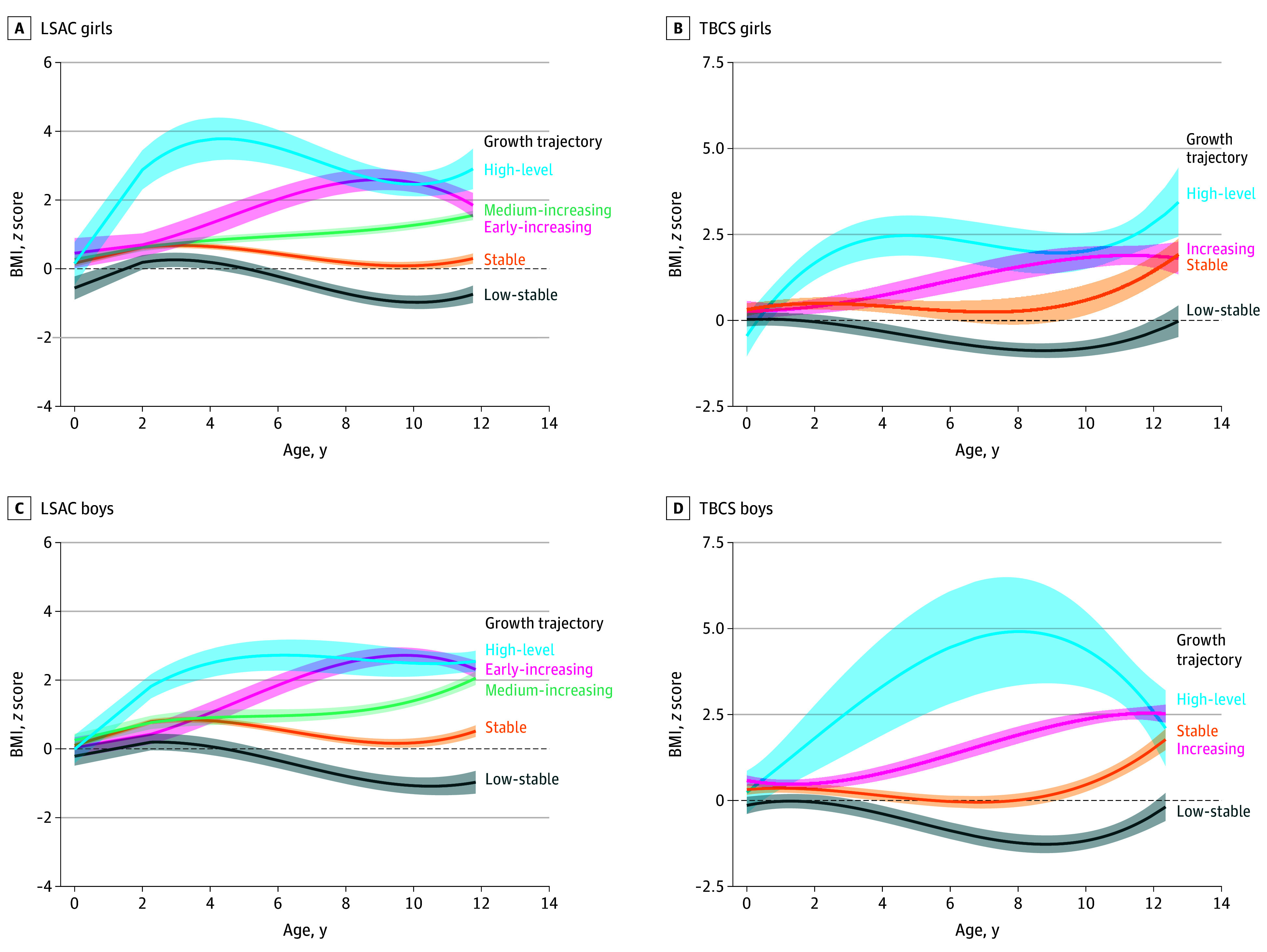
Line Graphs of Childhood Body Mass Index (BMI) *z* Scores of Trajectory Groups by Age Five distinct trajectory patterns among 1723 boys (C) and 1631 girls (A) in the Longitudinal Study of Australian Children (LSAC) and 4 distinct trajectory patterns among 542 boys (D) and 563 girls (B) in the Tianjin Birth Cohort Study (TBCS), modeled by a latent class growth mixed model. BMI was calculated as weight in kilograms divided by height in meters squared.

Five distinct trajectory patterns of BMI *z* scores were identified in the LSAC and 4 in the TBCS (Figure 1). Compared with those in the stable group (Table 1), girls in almost all of the high-level or increasing trajectory groups in both the LSAC and the TBCS were younger at pubertal onset (from β = −0.36 [95% CI, −0.66 to −0.07] years to β = −1.51 [95% CI, −2.68 to −0.35] years) and were associated with increased risk of pubertal initiation (from hazard ratio [HR], 1.35 [95% CI, 1.04 to 1.74] to HR, 2.80 [95% CI, 1.69 to 4.63]), whereas an inverse association was observed in the low-stable groups of both cohorts. Similar patterns were found among boys of the LSAC but not for boys of the TBCS. These associations were robust in discrete-time survival models (model 2 in eTable 13 in Supplement 1), with a combined sample of all girls and LSAC boys, generating an HR from 1.15 (95% CI, 1.01 to 1.32) to 2.88 (95% CI, 1.85 to 4.49).

**Table 1.** Association of Different Childhood Body Mass Index *z* Scores of Trajectory Groups With Age at Pubertal Onset and Risk of Pubertal Onset at Each Age^a^

Trajectory group variable	β or HR (95% CI)^b^			
	Girls		Boys	
	Model 1^c^	Model 2^d^	Model 1^c^	Model 2^d^
Age at pubertal onset, y				
LSAC				
High-level	**−1.64 (−2.78 to −0.49)**	**−1.51 (−2.68 to −0.35)**	**−0.82 (−1.32 to −0.31)**	**−0.67 (−1.19 to −0.16)**
Early-increasing	−1.14 (−1.69 to −0.58)	−1.22 (−1.81 to −0.64)	−0.51 (−0.91 to −0.11)	−0.47 (−0.89 to −0.05)
Medium-increasing	−0.79 (−0.97 to −0.60)	−0.73 (−0.92 to −0.54)	−0.17 (−0.39 to 0.05)	−0.18 (−0.41 to 0.05)
Stable	1 [Reference]	1 [Reference]	1 [Reference]	1 [Reference]
Low-stable	0.67 (0.42 to 0.93)	0.70 (0.44 to 0.97)	0.51 (0.21 to 0.80)	0.53 (0.23 to 0.83)
TBCS				
High-level	−0.44 (−0.99 to 0.12)	−0.42 (−0.98 to 0.14)	−0.13 (−1.26 to 0.99)	0.34 (−0.87 to 1.55)
Increasing	−0.33 (−0.62 to −0.05)	−0.36 (−0.66 to −0.07)	0.08 (−0.27 to 0.43)	0.13 (−0.23 to 0.49)
Stable	1 [Reference]	1 [Reference]	1 [Reference]	1 [Reference]
Low-stable	0.37 (0.13 to 0.61)	0.38 (0.13 to 0.62)	0.10 (−0.37 to 0.57)	0.09 (−0.39 to 0.57)
**Risk of pubertal onset at each age by the last round**				
LSAC				
High-level	3.09 (1.88 to 5.08)	2.80 (1.69 to 4.63)	1.37 (1.05 to 1.79)	1.28 (0.97 to 1.70)
Early-increasing	1.91 (1.44 to 2.53)	1.93 (1.44 to 2.59)	1.25 (0.99 to 1.57)	1.19 (0.94 to 1.52)
Medium-increasing	1.62 (1.45 to 1.81)	1.58 (1.41 to 1.78)	1.14 (1.00 to 1.30)	1.16 (1.01 to 1.32)
Stable	1 [Reference]	1 [Reference]	1 [Reference]	1 [Reference]
Low-stable	0.72 (0.62 to 0.85)	0.71 (0.60 to 0.84)	0.77 (0.65 to 0.92)	0.77 (0.64 to 0.92)
TBCS				
High-level	1.31 (0.83 to 2.06)	1.33 (0.84 to 2.13)	0.86 (0.38 to 1.94)	0.61 (0.25 to 1.51)
Increasing	1.29 (1.01 to 1.66)	1.35 (1.04 to 1.74)	0.96 (0.75 to 1.22)	0.93 (0.73 to 1.19)
Stable	1 [Reference]	1 [Reference]	1 [Reference]	1 [Reference]
Low-stable	0.75 (0.60 to 0.93)	0.75 (0.60 to 0.93)	0.91 (0.65 to 1.26)	0.92 (0.66 to 1.28)

Abbreviations: HR, hazard ratio; LSAC, Longitudinal Study of Australian Children; TBCS, Tianjin Birth Cohort Study.

^a^ Body mass index was calculated as weight in kilograms divided by height in meters squared.

^b^ β (95% CI) was used when the outcome was age at pubertal onset, and HR (95% CI) was used when the outcome was risk of pubertal onset at each age by the last round.

^c^ Null model.

^d^ Adjusted for maternal educational level, delivery mode, breastfeeding, fruit intake frequency, vegetable intake frequency (in both cohorts), parity, and children’s choice to spend free time (in the LSAC) and for maternal age at delivery and weekly frequency of vigorous exercise (in the TBCS).

### Association of CEA With Age at Pubertal Onset and Risk of Pubertal Onset at Each Age

Table 2 shows that among girls in both the LSAC and the TBCS, higher CE2 and ACE2 were associated with an earlier age at pubertal onset (from β = −0.04 [95% CI, −0.05 to −0.03] years to β = −0.85 [95% CI, −1.48 to −0.23] years), with a greater effect size after averaging. Results associated with CE1 were similar, although some results in the TBCS were nonsignificant. Consistent results were found in boys of the LSAC but not those of the TBCS.

**Table 2.** Association of Cumulative Exposures to Different Levels of Adiposity With Age at Pubertal Onset and Risk of Pubertal Onset at Each Age

Exposure	β or HR (95% CI)^a^			
	Girls		Boys	
	Model 1^b^	Model 2^c^	Model 1^b^	Model 2^c^
**Age at pubertal onset, y**				
LSAC				
Cumulative exposures to BMI *z *scores >1 to ≤2	−0.09 (−0.13 to −0.05)	−0.08 (−0.12 to −0.04)	−0.10 (−0.13 to −0.06)	−0.09 (−0.12 to −0.05)
Duration of BMI *z *scores >1 to ≤2	−0.06 (−0.09 to −0.03)	−0.05 (−0.08 to −0.03)	−0.05 (−0.08 to −0.02)	−0.05 (−0.08 to −0.02)
Average cumulative exposures to BMI *z *scores >1 to ≤2	−0.05 (−0.11 to 0.002)	−0.05 (−0.11 to 0.01)	−0.09 (−0.14 to −0.04)	−0.07 (−0.13 to −0.02)
Cumulative exposures to BMI *z *scores >2	−0.11 (−0.19 to −0.03)	−0.10 (−0.18 to −0.02)	−0.09 (−0.14 to −0.05	−0.08 (−0.12 to −0.03)
Duration of BMI *z *scores >2	−0.11 (−0.19 to −0.03)	−0.11 (−0.19 to −0.03)	−0.11 (−0.15 to −0.06)	−0.09 (−0.14 to −0.04)
Average cumulative exposures to BMI *z *scores >2	−0.91 (−1.52 to −0.30)	−0.85 (−1.48 to −0.23)	−0.86 (−1.23 to −0.48)	−0.73 (−1.12 to −0.35)
TBCS				
Cumulative exposures to BMI *z *scores >1 to ≤2	−0.03 (−0.09 to 0.02)	−0.03 (−0.09 to 0.02)	0.10 (0.04 to 0.16)	0.12 (0.05 to 0.18)
Duration of BMI *z *scores >1 to ≤2	−0.05 (−0.09 to 0.002)	−0.05 (−0.10 to 0.00)	0.01 (−0.06 to 0.09)	0.03 (−0.05 to 0.10)
Average cumulative exposures to BMI *z *scores >1 to ≤2	−0.04 (−0.12 to 0.04)	−0.02 (−0.11 to 0.06)	0.07 (−0.04 to 0.17)	0.09 (−0.02 to 0.20)
Cumulative exposures to BMI *z *scores >2	−0.04 (−0.05 to −0.03)	−0.04 (−0.05 to −0.03)	−0.004 (−0.02 to 0.009)	−0.001 (−0.01 to 0.01)
Duration of BMI *z *scores >2	−0.04 (−0.11 to 0.04)	−0.03 (−0.11 to 0.05)	0.17 (0.09 to 0.26)	0.20 (0.11 to 0.28)
Average cumulative exposures to BMI *z *scores >2	−0.21 (−0.32 to −0.10)	−0.21 (−0.32 to −0.10)	0.12 (−0.002 to 0.23)	0.15 (0.02 to 0.27)
**Risk of pubertal onset at each age by the last round**				
LSAC				
Cumulative exposures to BMI *z *scores >1 to ≤2	1.29 (1.17 to 1.43)^d^	1.27 (1.15 to 1.42)^d^	1.31 (1.11 to 1.53)^e^	1.33 (1.12 to 1.58)^e^
Duration of BMI *z *scores >1 to ≤2	1.29 (1.17 to 1.43)^d^	1.27 (1.15 to 1.42)^d^	1.17 (1.06 to 1.30)^d^	1.16 (1.04 to 1.29)^d^
Average cumulative exposures to BMI *z *scores >1 to ≤2	1.04 (1.02 to 1.06)	1.04 (1.01 to 1.06)	1.03 (1.01 to 1.06)	1.02 (0.99 to 1.05)
Cumulative exposures to BMI *z *scores >2	1.07 (1.04 to 1.11)	1.06 (1.03 to 1.10)	1.25 (1.09 to 1.43)^d^	1.21 (1.04 to 1.41)^d^
Duration of BMI *z *scores >2	1.28 (1.07 to 1.52)^d^	1.31 (1.09 to 1.58)^d^	1.31 (1.07 to 1.60)^e^	1.33 (1.07 to 1.65)^e^
Average cumulative exposures to BMI *z *scores >2	1.78 (1.37 to 2.32)	1.68 (1.29 to 2.19)	8.86 (1.71 to 45.83)^e^	9.69 (1.66 to 56.57)^e^
TBCS				
Cumulative exposures to BMI *z *scores >1 to ≤2	1.25 (1.04 to 1.51)^d^	1.27 (1.05 to 1.55)^d^	0.76 (0.61 to 0.95)^d^	0.73 (0.58 to 0.92)^d^
Duration of BMI *z *scores >1 to ≤2	1.25 (1.04 to 1.51)^d^	1.27 (1.05 to 1.55)^d^	1.00 (0.95 to 1.05)	0.99 (0.94 to 1.04)
Average cumulative exposures to BMI *z *scores >1 to ≤2	1.03 (0.97 to 1.09)	1.02 (0.96 to 1.09)	0.77 (0.61 to 0.96)^d^	0.74 (0.59 to 0.93)^d^
Cumulative exposures to BMI *z *scores >2	1.03 (1.02 to 1.04)	1.03 (1.02 to 1.04)	0.78 (0.62 to 0.99)^d^	0.95 (0.59 to 0.95)^d^
Duration of BMI *z *scores >2	1.03 (0.97 to 1.09)	1.03 (0.96 to 1.09)	0.89 (0.84 to 0.95)	0.88 (0.82 to 0.94)
Average cumulative exposures to BMI *z *scores >2	1.17 (1.07 to 1.28)	1.18 (1.07 to 1.29)	0.78 (0.62 to 0.99)^d^	0.75 (0.59 to 0.95)^d^

Abbreviations: BMI, body mass index (calculated as weight in kilograms divided by height in meters squared); HR, hazard ratio; LSAC, Longitudinal Study of Australian Children; TBCS, Tianjin Birth Cohort Study.

^a^ β (95% CI) was used when the outcome was age at pubertal onset, and HR (95% CI) was used when the outcome was risk of pubertal onset at each age by the last round.

^b^ Null model.

^c^ Adjusted for maternal educational level, delivery mode, breastfeeding, fruit intake frequency, vegetable intake frequency (in both cohorts), parity, and children’s choice to spend free time (in the LSAC) and for maternal age at delivery and weekly frequency of vigorous exercise (in the TBCS).

^d^ Variable exhibited a nonlinear association rather than a linear association. The nonlinear curves and corresponding *P *values are in eFigure 3 in Supplement 1. The effect size here indicates the zero-indicator effect.

^e^ Baseline HR (95% CI) for variables violating the proportional hazards assumption.

For risk of pubertal onset at each age, both linear and nonlinear associations were detected. CE1, along with its duration, was nonlinearly associated with risk of pubertal onset, showing an inverted U-shaped trend across both cohorts and both sexes (eFigure 3 in Supplement 1). CE2, its duration, and ACE2 were predominantly linearly associated with risk of pubertal onset and were factors associated with increased risk (Table 2), except for the reduced risk observed among boys of the TBCS. Notably, some results from boys of the LSAC showed a reduced risk, with the risk taking approximately 13 to 14 years to fully diminish (Table 2 and eTable 14 in Supplement 1). Similarly, discrete-time survival models in sensitivity analyses also identified CE1, CE2, their durations, and averaged estimates as factors associated with increased risk for pubertal onset at each age (model 2 in eTable 15 in Supplement 1).

### Stratified Analysis by Overall BMI Status

The associations of overall BMI status with the age at pubertal onset and risk of pubertal onset at each age were shown in eTable 16 in Supplement 1. For girls, BMI *z* scores at least once within the range of more than 1 to 2 or less were associated with an earlier age and increased risk of pubertal onset at each age in both cohorts, while the significant results of BMI *z* scores ever greater than 2 were only observed in the LSAC. For boys in the LSAC, results were similar to that of girls, whereas findings for boys in the TBCS were opposite. The stratified analysis examining the effect sizes of CEA by overall BMI status yielded findings similar to the total sample (eTable 17 in Supplement 1).

### BMI Increase and Sensitive Age at Pubertal Onset and Risk of Pubertal Onset at Each Age

**Figure 2.**
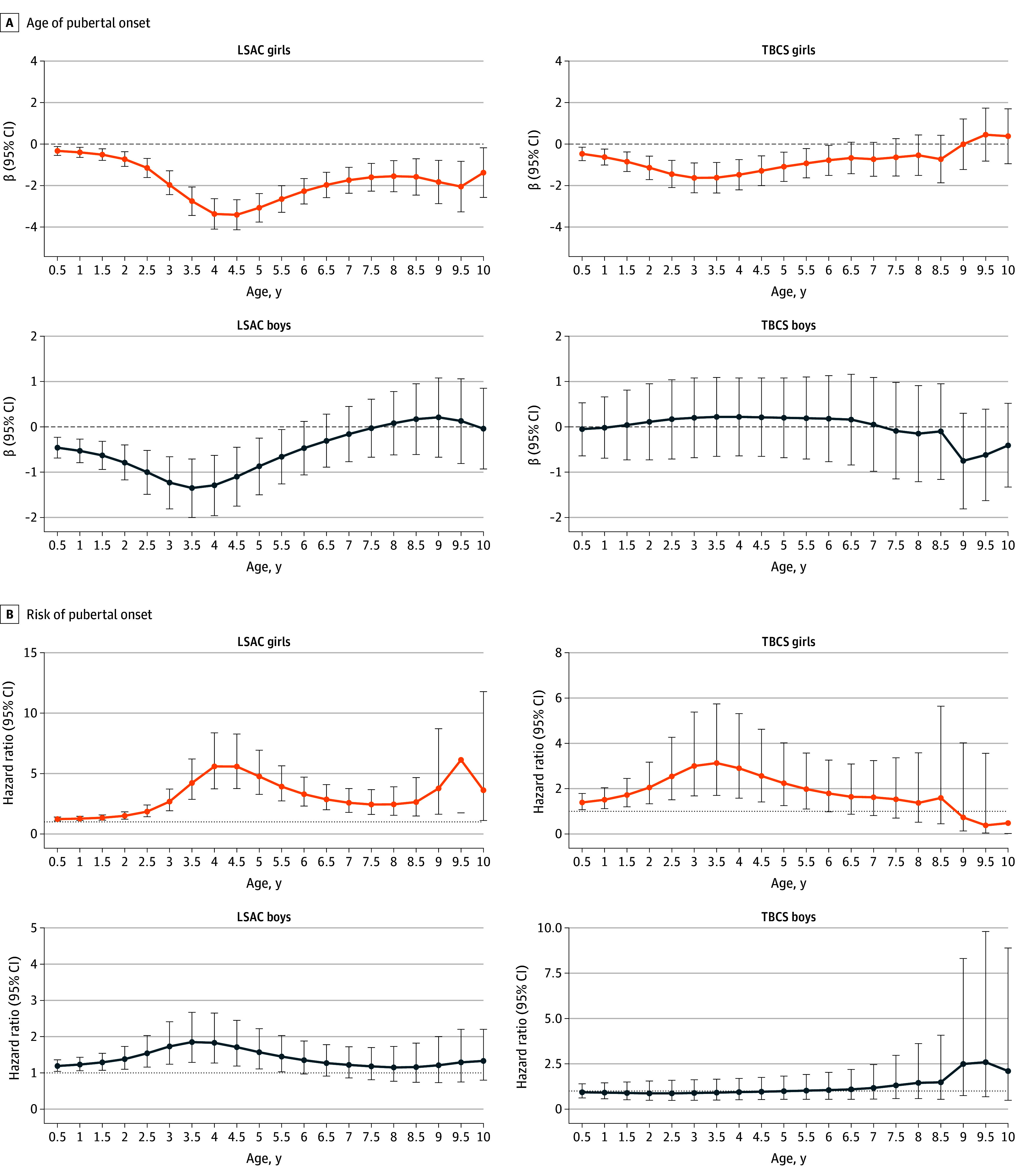
Line Graphs of Associations of Model Estimated Linear Slopes of Body Mass Index (BMI) *z* Scores by Age Outcomes of age at pubertal onset (A) and risk of pubertal onset at each age (B) among total boys and girls in the Longitudinal Study of Australian Children (LSAC) and the Tianjin Birth Cohort Study (TBCS). Models were adjusted for model estimated BMI *z* score level, maternal educational level, delivery mode, breastfeeding, fruit intake frequency, vegetable intake frequency (in both cohorts), parity, and children’s choice to spend free time (in the LSAC) and for maternal age at delivery and weekly frequency of vigorous exercise (in the TBCS). Some values in both sexes of TBCS were not shown because the sample size at these ages were so small that their intervals were too wide to display on the chart. BMI was calculated as weight in kilograms divided by height in meters squared.

Figure 2 presents the association of the rate of BMI increase at each age with the age at pubertal onset and with risk of pubertal onset at each age after adjustments, with the unadjusted model shown in eFigure 4 in Supplement 1. For girls, results from both cohorts showed similar patterns, with the greater effect size of pubertal onset at age 4.5 years (adjusted β = −3.41 [95% CI, −4.13 to −2.68]) and risk of pubertal onset at age 4.0 years (adjusted HR, 5.59 [95% CI, 3.73 to 8.37]) in the LSAC and greater effect size of pubertal onset at age 3.0 (adjusted β = −1.63 [95% CI, −2.35 to −0.91]) and risk of pubertal onset at age 3.5 years (adjusted HR, 3.13 [95% CI, 1.70 to 5.74]) in the TBCS. For boys, the greater effect size (adjusted β = −1.35 [95% CI, −2.00 to −0.71]) and risk (adjusted HR, 1.85 [95% CI, 1.29 to 2.67]) of pubertal onset were at age 3.5 years in the LSAC but were not observed in the TBCS. Similar patterns were found in both sexes among subgroups of distinct overall BMI status (eFigure 5 in Supplement 1).

Based on the aforementioned sensitive age identified (ages 3-4 years), Table 3 demonstrates that both CE1 and CE2 in the LSAC were associated with pubertal onset, with greater effect sizes (from β = −0.36 [95% CI, −0.65 to −0.06] years to β = −0.69 [95% CI, −1.28 to −0.09] years) and risk of pubertal onset (from HR, 1.30 [95% CI, 1.07 to 1.58] to 6.22 [95% CI, 1.38 to 28.00]) during the sensitive age window than outside of it. Similar results were observed in the TBCS although they were not significant. Sensitivity analyses using discrete-time survival models yielded consistent findings (model 2 in eTable 18 in Supplement 1).

**Table 3.** Association of Cumulative Exposure to Different Levels of Adiposity During and Outside of the Sensitive Ages of 3 to 4 Years With Age and Risk of Pubertal Onset at Each Age

Exposure	β or HR (95% CI)^a^			
	Girls		Boys	
	Model 1^b^	Model 2^c^	Model 1^b^	Model 2^c^
**Age at pubertal onset, y**				
LSAC				
Cumulative exposure to BMI *z *scores >1 to ≤2				
During the age period^d^	−0.56 (−0.88 to −0.24)	−0.50 (−0.82 to −0.18)	−0.41 (−0.70 to −0.13)	−0.36 (−0.65 to −0.06)
Outside of the age period	−0.09 (−0.13 to −0.04)	−0.08 (−0.13 to −0.04)	−0.10 (−0.14 to −0.07)	−0.09 (−0.13 to −0.06)
Cumulative exposure to BMI *z *scores >2				
During the age period^d^	−0.77 (−1.36 to −0.18)	−0.69 (−1.28 to −0.09)	−0.74 (−1.21 to −0.28)	−0.60 (−1.08 to −0.13)
Outside of the age period	−0.12 (−0.21 to −0.03)	−0.11 (−0.20 to −0.01)	−0.10 (−0.15 to −0.05)	−0.08 (−0.13 to −0.03)
TBCS				
Cumulative exposure to BMI *z *scores >1 to ≤2				
During the age period^d^	−0.19 (−0.68 to 0.30)	−0.15 (−0.65 to 0.36)	−0.70 (−1.50 to 0.10)	−0.49 (−1.31 to 0.33)
Outside of the age period	−0.04 (−0.09 to 0.02)	−0.03 (−0.10 to 0.03)	0.11 (0.05 to 0.17)	0.12 (0.06 to 0.19)
Cumulative exposure to BMI *z *scores >2				
During the age period^d^	−0.69 (−1.29 to −0.09)	−0.54 (−1.12 to 0.04)	−0.26 (−0.92 to 0.40)	−0.12 (−0.80 to 0.56)
Outside of the age period	−0.11 (−0.20 to −0.01)	−0.08 (−0.20 to 0.03)	0.05 (−0.01 to 0.12)	0.07 (0.01 to 0.14)
**Risk of pubertal onset at each age by the last round**				
LSAC				
Cumulative exposure to BMI *z *scores >1 to ≤2				
During the age period^d^	1.36 (1.13 to 1.64)	1.30 (1.07 to 1.58)	6.35 (1.54 to 26.11)^e^	6.22 (1.38 to 28.00)^e^
Outside of the age period	1.06 (1.03 to 1.08)	1.06 (1.03 to 1.08)	1.32 (1.11 to 1.56)^e^	1.34 (1.12 to 1.61)^e^
Cumulative exposure to BMI *z *scores >2				
During the age period^d^	1.65 (1.32 to 2.08)	1.58 (1.25 to 1.99)	1.33 (1.04 to 1.69)	1.24 (0.96 to 1.61)
Outside of the age period	1.08 (1.04 to 1.12)	1.07 (1.03 to 1.11)	1.30 (1.05 to 1.60)^e^	1.31 (1.05 to 1.65)^e^
TBCS				
Cumulative exposure to BMI *z *scores>1 to ≤2				
During the age period^d^	1.26 (0.81 to 1.96)	1.26 (0.80 to 1.97)	1.51 (0.90 to 2.52)	1.29 (0.77 to 2.17)
Outside of the age period	1.03 (0.98 to 1.08)	1.03 (0.99 to 1.08)	0.94 (0.90 to 0.98)	0.93 (0.87 0.97)
Cumulative exposure to BMI *z *scores >2				
During the age period^d^	1.55 (1.01 to 2.39)	1.50 (0.97 to 2.32)	1.12 (0.67 to 1.85)	0.98 (0.57 to 1.70)
Outside of the age period	1.08 (0.99 to 1.18)	1.08 (0.98 to 1.17)	0.95 (0.90 to 1.00)	0.93 (0.88 to 0.99)

Abbreviations: BMI, body mass index (calculated as weight in kilograms divided by height in meters squared); HR, hazard ratio; LSAC, Longitudinal Study of Australian Children; TBCS, Tianjin Birth Cohort Study.

^a^ β (95% CI) was used when the outcome was age at pubertal onset, and HR (95% CI) was used when the outcome was risk of pubertal onset at each age by the last round.

^b^ Null model.

^c^ Adjusted for maternal educational level, delivery mode, breastfeeding, fruit intake frequency, vegetable intake frequency (in both cohorts), parity, and children’s choice to spend free time (in the LSAC) and for maternal age at delivery and weekly frequency of vigorous exercise (in the TBCS).

^d^ The identified sensitive age window of 3 to 4 years.

^e^ Baseline HR (95% CI) for variables violating the proportional hazards assumption.

## Discussion

Based on 2 relatively homogenous populations of children from different countries, this cohort study found largely consistent findings, namely that high-level or increasing prepubertal growth trajectories and greater CEA were associated with earlier pubertal onset and higher risk of pubertal onset at each age, except for boys in the TBCS. Moreover, the ages of 3 to 4 years were identified as a potential sensitive period for BMI increase associated with early pubertal timing.

Our findings of BMI trajectories among girls are consistent with previous literature,^15,16,29,30^ but findings in boys are less consistent.^15,29,30^ Notably, the advancement of 1.51 years observed in girls with the highest BMI trajectory in the LSAC may extend the window of endogenous estrogen exposure, which has been linked to a higher breast cancer incidence, highlighting the importance of early weight management.^31^ Given that BMI trajectory only indicated children’s BMI level, the combination of both the degree and duration of adiposity was considered to account for CEA. Previous research on CEA has focused on diabetes, cardiovascular risk factors, and cancer in adulthood.^17,18,19,32^ To our knowledge, this is the first pediatric study to calculate CEA across the first decade of life in relation to pubertal timing.

As summarized in a review, few previous longitudinal pediatric studies have distinguished between a group exclusively with overweight and a group exclusively with obesity; even among these few studies, most failed to account for the duration of overweight and obese statuses.^33^ Our classification of adiposity levels was based on BMI *z* score cutoffs of 1 and 2, which are the most commonly established thresholds for defining overweight and obesity in children. By integrating the cumulative exposure approach, this longitudinal study differentiated the outcomes of different adiposity levels, thereby providing insights for this field.

Moreover, another effort we made to distinguish adiposity levels in such a longitudinal study was identifying the overall BMI status, which evaluated whether a child’s BMI *z* scores ever fell into the range of more than 1 to 2 or less or exceeded 2 during the follow-up period, even once, irrespective of whether a child’s BMI returned to normal later. The results, together with findings of CEA, indicate that both greater CEA and BMI *z* scores ever exceeding 2 were factors associated with increased risk for earlier pubertal timing; however, an inverse association was observed among boys of the TBCS, consistent with several studies reporting opposite effects of obesity and pubertal timing in boys.^34,35,36,37^ For example, Lee et al^34^ found that boys with obesity matured later than those with normal weight, and Crocker et al^36^ and Wang^37^ both reported a negative association between boys’ obesity and pubertal maturation.

The observed sexual dimorphisms in the TBCS could be attributed to both cohort-specific characteristics and biologic mechanisms. Beyond race and ethnicity composition (ie, children in Australia and China), cohort-specific characteristics such as pubertal assessment intervals (every 2 years in the LSAC vs every 6 months in the TBCS), coverage age period (a lower proportion of boys who had entered puberty in the TBCS), and limited sample size may account for the discrepant results. In contrast, for girls, both cohorts covered similar and appropriate age ranges to capture pubertal onset, which may have led to consistent findings. For biologic mechanisms, Lee et al^34^ speculated that greater estrogen production in boys with high-level adiposity may suppress the pubertal process. Leptin may also contribute to the sex difference in pubertal timing through its activation of kisspeptin neurons in girls with obesity but not in boys and its suppression by testosterone in boys.^38,39^

Most existing literature exploring the notion of the sensitive period of rapid growth on pubertal timing has drawn conclusions by comparing changes in growth indicators over several arbitrarily defined time intervals, resulting in inconsistent conclusions across studies.^20,40^ Therefore, to our knowledge, this is the first pediatric study that identified a sensitive age based on age-specific rates of BMI increase across the first decade. The identified ages 3 to 4 years could be supported by pediatric studies demonstrating that the excess weight in children with obesity has largely accumulated in preschool years (eg, by age 5 years or between ages 2 and 6 years).^22,41^ We also speculated that children who experience accelerated weight gain during this window may undergo adiposity rebound earlier than 3 years of age, a well-established predictor for earlier pubertal onset.

### Limitations

This study has limitations. First, information about pubertal onset was reported by parents rather than assessment by trained clinicians. It may thus be subject to measurement error, especially for the assessment of breast development, given that thelarche can be difficult to differentiate from lipomastia. Validation using clinical assessment in subsamples would be valuable. Second, as BMI does not distinguish fat from lean mass, our use of BMI and BMI *z* scores as adiposity proxies meant that the findings may not have directly reflected adiposity’s association with pubertal onset. Third, despite the recommendation of weight for length for children under 2 years of age, we used BMI throughout to ensure continuity, supported by studies showing high agreement between weight for length and BMI and comparable associations with later cardiometabolic outcomes.^42,43^ Fourth, the younger age of boys and the small sample size in the TBCS could be addressed by access to data from an older cohort in the future.^4^ Fifth, we were unable to account for some potential confounding factors, such as maternal age at menarche and exposure to endocrine-disrupting compounds, as these were not collected in either study. Sixth, several analytic limitations should be considered. Interval regression models only captured event occurrence within predefined intervals, which may have led to loss of precise timing information. Cox proportional hazards regression models relied on the proportional hazards assumption and were sensitive to tied event times. Discrete-time survival models required dividing the time scale into intervals, which may have been sensitive to the selection of interval width. Seventh, the 2-stage analytic approach may have introduced misclassification bias. Eighth, using the same dataset for both effect estimation of cumulative exposure and sensitive window identification may have led to overfitting and optimistic effect sizes.

## Conclusions

In this cohort study, greater CEA among children was associated with an earlier age and increased likelihood of pubertal onset, highlighting the importance of considering minimizing rapid prepubertal weight gain. Specifically, the findings of an apparently sensitive period at 3 to 4 years of age suggest that preschool years may be a priority age window for preventive intervention. These findings may have implications for health professionals, parents, and caregivers.

## Notes

Supplement 1.
eMethods

eReferences
**eFigure 1.** The Selection Process of the Final Analytic Sample of LSAC and TBCS **eTable 1.** Prevalence of Different Levels of Adiposity and Incidence of Pubertal Onset in LSAC **eTable 2.** Prevalence of Different Levels of Adiposity and Incidence of Pubertal Onset in TBCS **eTable 3.** Latent Class Growth Mixture Models Results of Model Fitting Process in Girls in LSAC **eTable 4.** Parameter Estimates for the Best-Fitting 4-Class Cubic Latent Class Growth Mixture Model of Girls in LSAC **eTable 5.** Latent Class Growth Mixture Models Results of Model Fitting Process in Girls in TBCS **eTable 6.** Parameter Estimates for the Best-Fitting 4-Class Cubic Latent Class Growth Mixture Model of Girls in TBCS **eTable 7.** Latent Class Growth Mixture Models Results of Model Fitting Process in Boys in LSAC **eTable 8.** Parameter Estimates for the Best-Fitting 4-Class Cubic Latent Class Growth Mixture Model of Boys in LSAC **eTable 9.** Latent Class Growth Mixture Models Results of Model Fitting Process in Boys in TBCS **eTable 10.** Parameter Estimates for the Best-Fitting 4-Class Cubic Latent Class Growth Mixture Model of Boys in TBCS **eFigure 2.** Examples of Calculation of the Cumulative Exposures to BMI *z *Scores Within (1,2] (Filled With Blue) and Cumulative Exposures to BMI *z* Scores >2 (Filled With Yellow) Derived From the BMI *z* Score Trajectories of 3 Random Children **eTable 11.** Tests of Proportional Hazards Assumption for Each Exposure Variable **eTable 12.** Characteristics of Participants by Sex **eTable 13.** Sensitivity Analyses of Childhood BMI *z* Score Trajectory Groups Associated With Risk of Pubertal Onset Based on a Discrete-Time Survival Model, With Comparison With Primary Survival Analyses **eFigure 3.** Nonlinear Associations Between Cumulative Exposures to Different Levels of Adiposity and Risk of Pubertal Onset **eTable 14.** Time-Varying Effects for Exposures Violating the Proportional Hazards Assumption (Only Observed in Boys of LSAC) **eTable 15.** Sensitivity Analyses of Association of Cumulative Exposures to Different Levels of Adiposity With Risk of Pubertal Onset Based on Discrete-Time Survival Model, With Comparison With Primary Survival Analyses **eTable 16.** Association of Overall BMI Status With Age and Risk of Pubertal Onset **eTable 17.** Association of Cumulative Exposures to Different Levels of Adiposity With Age and Risk of Pubertal Onset Stratified by Overall BMI Status **eFigure 4.** Associations of Model Estimated Linear Slopes of BMI *z* Scores by Age With Age and Risk of Pubertal Onset Without Adjustment **eFigure 5.** Associations of Model Estimated Linear Slopes of BMI *z* Scores by Age With Age and Risk of Pubertal Onset Stratified by Total BMI Status **eTable 18.** Sensitivity Analyses of Association of Cumulative Exposure to Different Levels of Adiposity During and Outside of the Sensitive Ages of 3 to 4 Years With Risk of Pubertal Onset Based on Discrete-Time Survival Model, With Comparison With Primary Survival Analyses

Supplement 2.
Data Sharing Statement
