# Supplementary material for: Prepubertal Growth Trajectory and Pubertal Onset
Source: JAMA Netw Open. 2026 Jun 9;9(6):e2617435. doi: 10.1001/jamanetworkopen.2026.17435 (PMC13250710; doi:10.1001/jamanetworkopen.2026.17435)
Supplement: Supplement 1. — eMethods eReferences eFigure 1. The Selection Process of the Final Analytic Sample of LSAC and TBCS eTable 1. Prevalence of Different Levels of Adiposity and Incidence of Pubertal Onset in LSAC eTable 2. Prevalence of Different Levels of Adiposity and Incidence of Pubertal Onset in TBCS eTable 3. Latent Class Growth Mixture Models Results of Model Fitting Process in Girls in LSAC eTable 4. Parameter Estimates for the Best-Fitting 4-Class Cubic Latent Class Growth Mixture Model of Girls in LSAC eTable 5. Latent Class Growth Mixture Models Results of Model Fitting Process in Girls in TBCS eTable 6. Parameter Estimates for the Best-Fitting 4-Class Cubic Latent Class Growth Mixture Model of Girls in TBCS eTable 7. Latent Class Growth Mixture Models Results of Model Fitting Process in Boys in LSAC eTable 8. Parameter Estimates for the Best-Fitting 4-Class Cubic Latent Class Growth Mixture Model of Boys in LSAC eTable 9. Latent Class Growth Mixture Models Results of Model Fitting Process in Boys in TBCS eTable 10. Parameter Estimates for the Best-Fitting 4-Class Cubic Latent Class Growth Mixture Model of Boys in TBCS eFigure 2. Examples of Calculation of the Cumulative Exposures to BMI z Scores Within (1,2] (Filled With Blue) and Cumulative Exposures to BMI z Scores >2 (Filled With Yellow) Derived From the BMI z Score Trajectories of 3 Random Children eTable 11. Tests of Proportional Hazards Assumption for Each Exposure Variable eTable 12. Characteristics of Participants by Sex eTable 13. Sensitivity Analyses of Childhood BMI z Score Trajectory Groups Associated With Risk of Pubertal Onset Based on a Discrete-Time Survival Model, With Comparison With Primary Survival Analyses eFigure 3. Nonlinear Associations Between Cumulative Exposures to Different Levels of Adiposity and Risk of Pubertal Onset eTable 14. Time-Varying Effects for Exposures Violating the Proportional Hazards Assumption (Only Observed in Boys of LSAC) eTable 15. Sensitivity Analyses of Association of Cumu [file jamanetwopen-e2617435-s001.pdf]

Supplemental Online Content

Deng R, Li W, Duan J, et al. Prepubertal growth trajectory and pubertal onset. *JAMA Netw Open*. 2026;9(6):e2617435. doi:10.1001/jamanetworkopen.2026.17435

eMethods
eReferences
eFigure 1. The Selection Process of the Final Analytic Sample of LSAC and TBCS
eTable 1. Prevalence of Different Levels of Adiposity and Incidence of Pubertal Onset in LSAC
eTable 2. Prevalence of Different Levels of Adiposity and Incidence of Pubertal Onset in TBCS
eTable 3. Latent Class Growth Mixture Models Results of Model Fitting Process in Girls in LSAC
eTable 4. Parameter Estimates for the Best-Fitting 4-Class Cubic Latent Class Growth Mixture Model of Girls in LSAC
eTable 5. Latent Class Growth Mixture Models Results of Model Fitting Process in Girls in TBCS
eTable 6. Parameter Estimates for the Best-Fitting 4-Class Cubic Latent Class Growth Mixture Model of Girls in TBCS
eTable 7. Latent Class Growth Mixture Models Results of Model Fitting Process in Boys in LSAC
eTable 8. Parameter Estimates for the Best-Fitting 4-Class Cubic Latent Class Growth Mixture Model of Boys in LSAC
eTable 9. Latent Class Growth Mixture Models Results of Model Fitting Process in Boys in TBCS
eTable 10. Parameter Estimates for the Best-Fitting 4-Class Cubic Latent Class Growth Mixture Model of Boys in TBCS
eFigure 2. Examples of Calculation of the Cumulative Exposures to BMI *z* Scores Within (1,2] (Filled With Blue) and Cumulative Exposures to BMI *z* Scores >2 (Filled With Yellow) Derived From the BMI *z* Score Trajectories of 3 Random Children
eTable 11. Tests of Proportional Hazards Assumption for Each Exposure Variable
eTable 12. Characteristics of Participants by Sex
eTable 13. Sensitivity Analyses of Childhood BMI *z* Score Trajectory Groups Associated With Risk of Pubertal Onset Based on a Discrete-Time Survival Model, With Comparison With Primary Survival Analyses
eFigure 3. Nonlinear Associations Between Cumulative Exposures to Different Levels of Adiposity and Risk of Pubertal Onset
eTable 14. Time-Varying Effects for Exposures Violating the Proportional Hazards Assumption (Only Observed in Boys of LSAC)
eTable 15. Sensitivity Analyses of Association of Cumulative Exposures to Different Levels of Adiposity With Risk of Pubertal Onset Based on Discrete-Time Survival Model, With Comparison With Primary Survival Analyses
eTable 16. Association of Overall BMI Status With Age and Risk of Pubertal Onset
eTable 17. Association of Cumulative Exposures to Different Levels of Adiposity With Age and Risk of Pubertal Onset Stratified by Overall BMI Status

© 2026 Deng R et al. *JAMA Network Open*.

eFigure 4. Associations of Model Estimated Linear Slopes of BMI *z* Scores by Age With Age and Risk of Pubertal Onset Without Adjustment
eFigure 5. Associations of Model Estimated Linear Slopes of BMI *z* Scores by Age With Age and Risk of Pubertal Onset Stratified by Total BMI Status
eTable 18. Sensitivity Analyses of Association of Cumulative Exposure to Different Levels of Adiposity During and Outside of the Sensitive Ages of 3 to 4 Years With Risk of Pubertal Onset Based on Discrete-Time Survival Model, With Comparison With Primary Survival Analyses

This supplemental material has been provided by the authors to give readers additional information about their work.

eMethods

BMI measurements

In LSAC, trained interviewers took all anthropometric measurements at all waves of data collection. Detailed information has been reported elsewhere.[1] In TBCS, child's length/height was measured to the nearest 0.1 centimeter using a recumbent length stadiometer (YSC-2, Beijing Guowangxingda, China) in infancy and a portable stadiometer during childhood. Weight was measured to the nearest 0.01 kilogram (kg) with children barefoot and in light clothes using a digital scale (TCS-60, Tianjin Weighing Apparatus Co., China).

Pubertal Development Scale (PDS)

The PDS avoids the use of explicit pictures or drawings that may be considered inappropriate in certain settings, thus making it suitable for large epidemiology studies. The PDS consists of five items that assess growth of body hair, height, skin changes (especially pimples) in both boys and girls, as well as age at menarche and breast growth in girls, and deepening-voice and facial hair growth in boys. Based on the total scores obtained by summing specific items, participants were classified into having initiated puberty or not.

BMI trajectory groups

© 2026 Deng R et al. *JAMA Network Open*.

The latent class trajectories of BMI z scores in the latent class growth mixed model (LCGMM) were specified as a function of centralized age (based on the mean age: 3.54 years for both sexes in TBCS, 5.70 years for boys and 5.69 years for girls in LSAC). Multiple LCGMMs with different trajectory patterns including linear and non-linear parameters were explored. First, with the same starting values calculated from one-group model, Repeated trajectory fittings were performed to identify the latent classes by changing the number of groups from two to five, with the same starting values calculated from one-group model. Then, to avoid convergence towards local maxima, LCGMMs with two to five classes were also then performed several times separately with different sets of random starting values to avoid convergence towards local maxima. The optimal model was selected by the following criteria: (1) Bayesian information criterion decreased at least 20; (2) High AvePP (> 0.65); and (3) High mean posterior probabilities (> 0.7). The best-fitting cubic model was described as: [2]

$$BMI_i = (f_{0g} + r_{0gi}) + (f_{1g} + r_{1gi}) \times age + (f_{2g} + r_{2gi}) \times age^2 + (f_{3g} + r_{3gi}) \times age^3$$

where individual "i" belongs to group "g", f = (f_{0g}+ f_{1g}+ f_{2g} + f_{3g}) is the vector of fixed-effect estimates in the group "g", r = (r_{0gi}+ r_{1gi}+ r_{2gi} + r_{3gi}) is the vector of random-effect estimates of the individual "i" in group "g".

Cumulative exposure to different levels of adiposity

The estimates of the cubic curve parameters computed by LCGMMs include fixed-effect parameters (for each group), and random-effect

© 2026 Deng R et al. *JAMA Network Open*.

parameters (for each child). The random-effect coefficients quantify the deviation of each individual's observed values from the group-level fixed-effect estimates. Individual curve parameters for each child were then generated by summing the fixed-effect parameters (vector f in the formula above) and random-effect parameters (vector r in the formula above).

Association analysis

Interval regression models were used to assess outcome of age at pubertal onset, with residual normality assessed by comparing the Turnbull-estimated empirical cumulative distribution against its theoretical counterpart. The outcome of age at pubertal onset was treated as interval-censored data, including left-censored, right-censored, interval-, and exact-observation data, determined by the wave at which puberty was reported and the preceding wave.

Cox proportional hazards models were used to assess outcome of occurrence of pubertal onset. The survival time was defined as the children's age at pubertal onset, which was assigned on an individual basis and as follows: the age at the first round for children who had already entered puberty at baseline and those with new-onset puberty during follow-up (with right-censored method applied to interval-censored data), and the age at last round for those who had not entered puberty by the end of the study. We assessed the proportional hazards assumption using Schoenfeld residuals (eTable 11). To address covariates that violated the proportional hazards assumption, we employed time-dependent Cox models by

© 2026 Deng R et al. *JAMA Network Open*.

treating them as time-dependent covariates and incorporating a time-by-covariate interaction term.[3-5] Baseline HRs with 95% CIs were reported in the primary results separately, while time-varying effects were presented in the supplementary material.

To address concerns regarding the proportional hazards assumption and ties in the Cox proportional hazards models, a sensitivity analysis using discrete-time survival models with a complementary log-log link function was further conducted. This approach divided the time scale into intervals and included them as categorical covariates in the models, which means it does not rely on the proportional hazards assumption. Given that pubertal onset rarely occurs before age 6 years, the time scale was divided into half-year intervals from 6 to 18 years (left truncation at 6 years).

Nonlinearity between CEA and risk of pubertal onset at each age was evaluated by the restricted cubic spline model with four knots (5th, 35th, 65th and 95th). For variables with significant nonlinearity, estimates of zero-indicator-effect was provided in tables since these variables followed zero-inflated skewed distribution.

© 2026 Deng R et al. *JAMA Network Open*.

eReferences

1. Mohal J LC, Gasser C, Howell L, Renda J, Jessup K, Daraganova G. Growing Up in Australia: The Longitudinal Study of Australian Children – Data User Guide. Australian Institute of Family Studies 2020.
2. Zhang T, Whelton PK, Xi B, et al. Rate of change in body mass index at different ages during childhood and adult obesity risk. *Pediatr Obes*. 2019;14(7):e12513. doi:10.1111/ijpo.12513
3. Liu Y, Gong Y, Zhu XZ, et al. Effect of adjuvant carboplatin intensified chemotherapy versus standard chemotherapy on survival in women with high risk, early stage, triple negative breast cancer (CITRINE): randomised, open label phase 3 trial. *BMJ*. 2025;391:e085457. doi:10.1136/bmj-2025-085457
4. Zeng Z, Gao Y, Li J, et al. Violations of proportional hazard assumption in Cox regression model of transcriptomic data in TCGA pan-cancer cohorts. *Comput Struct Biotechnol J*. 2022;20:496-507. doi:10.1016/j.csbj.2022.01.004
5. Jiang N, Wu Y, Li C. Limitations of using COX proportional hazards model in cardiovascular research. Cardiovasc Diabetol. 2024;23(1):219. doi:10.1186/s12933-024-02302-2

© 2026 Deng R et al. *JAMA Network Open*.

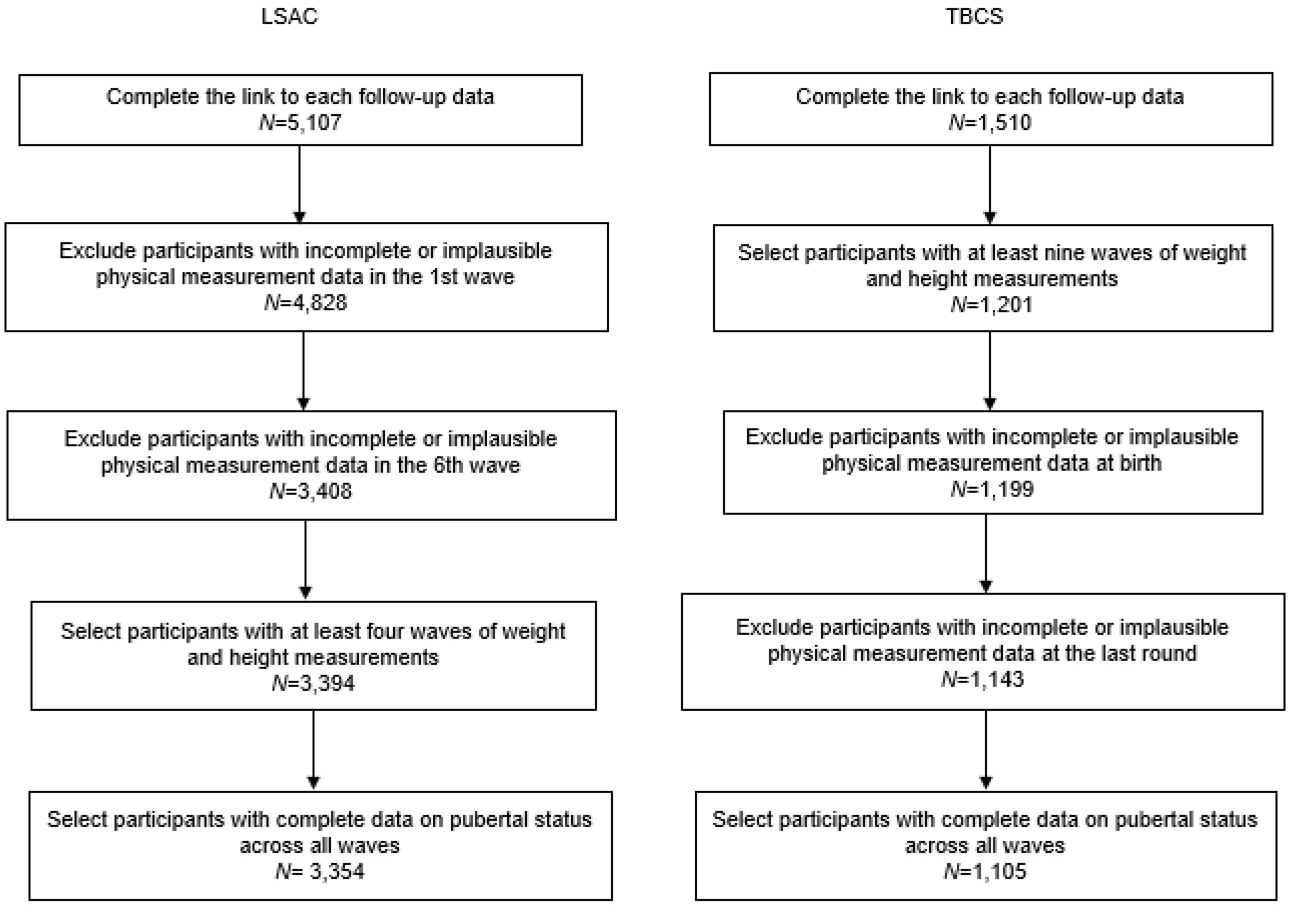

eFigure 1. The Selection Process of the Final Analytic Sample of LSAC and TBCS

Note: LSAC, longitudinal study of Australian children; TBCS, Tianjin birth cohort study

© 2026 Deng R et al. *JAMA Network Open*.

eTable 1. Prevalence of Different Levels of Adiposity and Incidence of Pubertal Onset in LSAC

	Girls					Boys				
	N	Age	Prevalence of BMI z scores within (1,2]	Prevalence of BMI Z scores >2	Incidence of pubertal onset	N	Age	Prevalence of BMI z scores within (1,2]	Prevalence of BMI z scores >2	Incidence of pubertal onset
Wave 1	1631	0	16.31%	3.25%	—	1723	0	15.61%	3.25%	—
Wave 2	1592	2.81±0.23	30.02%	10.64%	—	1678	2.82±0.24	30.94%	13.50%	—
Wave 3	1591	4.78±0.23	20.97%	6.99%	—	1686	4.79±0.23	29.30%	9.41%	—
Wave 4	1593	6.81±0.29	20.70%	6.01%	—	1687	6.81±0.29	18.77%	9.86%	—
Wave 5	1605	8.88±0.30	21.05%	8.38%	39.0%	1686	8.88±0.30	19.48%	12.83%	—
Wave 6	1631	10.87±0.33	22.87%	8.58%	84.1%	1723	10.88±0.34	20.89%	15.03%	38.8%
Wave 7	1631	12.92±0.36	20.42%	6.56%	97.0%	1723	12.92±0.36	17.70%	9.87%	74.06%
Wave 8	1631	14.83±0.69	18.03%	5.70%	98.65%	1723	14.83±0.53	14.05%	8.13%	91.41%

—, survey for the corresponding item not conducted. BMI, body mass index.

© 2026 Deng R et al. *JAMA Network Open*.

eTable 2. Prevalence of Different Levels of Adiposity and Incidence of Pubertal Onset in TBCS

	Girls					Boys				
	**N**	**Age**	**Prevalence of BMI z scores within (1,2]**	**Prevalence of BMI z scores >2**	**Incidence of pubertal onset**	**N**	**Age**	**Prevalence of BMI z scores within (1,2]**	**Prevalence of BMI z scores >2**	**Incidence of pubertal onset**
Wave 1	563	0	13.32%	1.60%	—	542	0	17.90%	2.40%	—
Wave 2	563	0.28±0.05	22.56%	5.15%	—	542	0.28±0.05	24.72%	9.23%	—
Wave 3	561	0.53±0.06	17.71%	7.16%	—	535	0.53±0.05	22.62%	7.10%	—
Wave 4	522	0.71±0.07	19.53%	6.37%	—	491	0.71±0.07	22.92%	8.33%	—
Wave 5	84	0.76±0.11	5.97%	13.43%	—	94	0.74±0.10	22.67%	6.67%	—
Wave 6	558	1.13±0.20	21.58%	6.29%	—	539	1.14±0.20	27.24%	7.28%	—
Wave 7	446	1.52±0.07	21.76%	8.80%	—	430	1.52±0.07	20.24%	8.67%	—
Wave 8	508	2.13±0.21	14.76%	5.12%	—	496	2.11±0.18	16.16%	5.05%	—
Wave 9	275	2.55±0.10	14.34%	1.84%	—	285	2.56±0.10	14.44%	4.58%	—
Wave 10	234	3.49±0.26	14.10%	3.85%	—	221	3.46±0.27	14.48%	4.52%	—
Wave 11	282	4.48±0.28	13.52%	1.78%	—	277	4.50±0.30	13.77%	7.25%	—
Wave 12	269	5.45±0.28	16.36%	3.35%	—	242	5.44±0.28	15.29%	9.09%	—
Wave 13	557	7.71±0.61	16.34%	9.69%	10.8%	538	7.73±0.61	17.66%	21.56%	5.2%
Wave 13a	563	—	—	—	21.7%	542	—	—	—	11.5%

© 2026 Deng R et al. *JAMA Network Open*.

Wave 13b	563	—	—	—	41.0%	542	—	—	—	17.9%
Wave 13c	563	—	—	—	52.2%	542	—	—	—	24.4%
Wave 14	563	9.60±0.60	23.80%	13.85%	65.9%	542	9.63±0.61	19.37%	35.24%	48.2%
Wave 14a	563	—	—	—	79.2%	542	—	—	—	52.2%
Wave 15	563	10.62±0.60	24.16%	10.48%	80.9%	542	10.65±0.61	22.32%	32.29%	58.9%

—, survey for the corresponding item not conducted. BMI, body mass index.

© 2026 Deng R et al. *JAMA Network Open*.

eTable 3. Latent Class Growth Mixture Models Results of Model Fitting Process in Girls in LSAC

No. of class	Polynomial degree	BIC	Entropy	% Participants per class	AvePP	Proportion of AvePP > 70%
	Linear	26040.24	1.00	100	NA	NA
1	Quadratic	25038.68	1.00	100	NA	NA
	Cubic	24056.26	1.00	100	NA	NA
	Linear	24614.62	0.00	53.59/46.41	0.51/0.49	NA
2	Quadratic	23630.62	0.49	67.44/32.56	0.87/0.81	85.82/77.21
	Cubic	22650.58	0.51	26.3/73.7	0.77/0.88	66.43/87.60
	Linear	24600.62	0.69	10.18/88.53/1.29	0.68/0.89/0.67	40.96/88.78/38.10
3	Quadratic	23582.18	0.67	1.59/33.29/65.11	0.82/0.81/0.86	69.23/78.45/84.56
	Cubic	23903.51	0.98	1.47/98.04/0.49	0.86/0.99/0.94	79.17/99.75/87.5
	Linear	24610.8	0.73	10.12/0.25/87.61/2.02	0.67/0.94/0.88/0.66	41.21/100/87.19/39.39
4	Quadratic	23605.06	0.54	9.38/56.77/1.53/32.31	0.67/0.69/0.86/0.79	38.56/49.14/84/71.54
	Cubic	22613.8	0.68	33.29/1.1/3.62/61.99	0.75/0.84/0.75/0.85	67.03/72.22/55.93/80.61
	Linear	24632.25	0.64	51.56/39.73/0.31/7.05/1.35	0.71/0.77/0.87/0.76/0.72	58.98/68.06/80/60.00/50.00
5	Quadratic	23614.94	0.52	12.39/56.41/16.25/0.98/13.98	0.67/0.68/0.65/0.94/0.64	41.09/48.59/38.11/93.75/35.96
	**Cubic**	**22627.53**	**0.71**	**29.98/54.69/0.98/3.19/11.16**	**0.81/0.82/0.88/0.82/0.77**	**75.05/82.74/75.00/73.08/63.74**

No. class, latent class number of the model; BIC, the Bayesian information Criterion; AvePP, Average posterior probabilities; NA, not applicable.

© 2026 Deng R et al. *JAMA Network Open*.

eTable 4. Parameter Estimates for the Best-Fitting 4-Class Cubic Latent Class Growth Mixture Model of Girls in LSAC

Parameters	Class	Fixed effect		Random effect: variance-covariance matrix
		Estimated value	*P*	
Intercept	Class 1	0.93	<0.001	σ^2_{int} = 0.484
	Class 2	0.48	<0.001	
	Class 3	3.60	<0.001	
	Class 4	1.92	<0.001	
	Class 5	-0.14	0.133	
Linear	Class 1	0.06	<0.001	$\sigma^2_{linear\ slope}$ = 0.008
	Class 2	-0.13	<0.001	Cov $_{(linear,\ intercept)}$ = -0.05
	Class 3	-0.25	<0.001	
	Class 4	0.34	<0.001	
	Class 5	-0.25	<0.001	
Quadratic	Class 1	-0.002	0.313	$\sigma^2_{quadratic\ slope}$ = 0.0003
	Class 2	-0.01	<0.001	Cov $_{(quadratic,\ intercept)}$ = -0.010
	Class 3	-0.07	<0.001	Cov $_{(quadratic,\ linear)}$ = 0.001
	Class 4	-0.02	<0.001	
	Class 5	-0.02	<0.001	
Cubic	Class 1	0.002	0.003	$\sigma^2_{cubic\ slope}$ = 2×10^{-5}
	Class 2	0.004	<0.001	Cov $_{(cubic,\ intercept)}$ = 0.002
	Class 3	0.01	<0.001	Cov $_{(cubic,\ linear)}$ = -0.0003
	Class 4	-0.01	<0.001	Cov $_{(cubic,\ quadratic)}$ = -0.0001
	Class 5	0.01	<0.001	RSE=0.005

RSE, residual standard error.

© 2026 Deng R et al. *JAMA Network Open*.

eTable 5. Latent Class Growth Mixture Models Results of Model Fitting Process in Girls in TBCS

No. of class	Polynomial degree	BIC	Entropy	% Participants per class	AvePP	Proportion of AvePP > 70%
1	Linear	16721.03	1.00	100	NA	NA
	Quadratic	16663.74	1.00	100	NA	NA
	Cubic	16504.57	1.00	100	NA	NA
2	Linear	16713.1	0.59	63.41/36.59	0.89/0.85	86.27/81.55
	Quadratic	16654.43	0.61	44.05/55.95	0.78/0.89	81.45/87.62
	Cubic	16502.48	0.62	60.92/39.08	0.91/0.86	88.92/80.91
3	Linear	16719.19	0.64	49.38/41.39/9.24	0.89/0.77/0.83	85.61/73.39/78.85
	Quadratic	16654.19	0.64	39.61/8.17/52.22	0.77/0.77/0.89	66.37/63.04/88.44
	Cubic	16511.10	0.66	14.74/39.43/45.83/	0.83/0.80/0.88	78.31/78.83/87.21
4	Linear	16732.96	0.68	28.95/35.17/28.24/7.64	0.85/0.76/0.82/0.87	80.98/71.21/80.50/86.05
	Quadratic	16661.6	0.72	49.02/42.45/5.86/2.66	0.90/0.79/0.74/0.76	91.30/72.80/63.64/53.33
	**Cubic**	**16522.88**	**0.65**	**20.6/3.91/37.48/38.01**	**0.79/0.78/0.85/0.72**	**69.83/72.73/78.67/60.75**
5	Linear	16748.2	0.60	12.79/24.87/30.37/25.22/6.75	0.56/0.76/0.67/0.80/0.84	13.89/62.86/46.20/74.65/78.95
	Quadratic	16680.45	0.69	2.84/45.83/40.5/8.88/1.95	0.65/0.85/0.77/0.74/0.77	43.75/82.56/70.18/56/63.64
	Cubic	16555.7	0.64	19.54/32.68/25.58/12.61/9.59	0.83/0.76/0.71/0.65/0.79	78.18/68.48/56.94/33.80/66.67

No. class, latent class number of the model; BIC, the Bayesian information Criterion; AvePP, Average posterior probabilities; NA, not applicable.

© 2026 Deng R et al. *JAMA Network Open*.

eTable 6. Parameter Estimates for the Best-Fitting 4-Class Cubic Latent Class Growth Mixture Model of Girls in TBCS

Parameters	Class	Fixed effect		Random effect: variance-covariance matrix
		Estimated value	*P*	
Intercept	Class 1	0.65	<0.001	σ^2_{int} = 0.482
	Class 2	2.34	<0.001	
	Class 3	-0.24	0.0356	
	Class 4	0.46	<0.001	
Linear	Class 1	0.18	<0.001	$\sigma^2_{linear\ slope}$ = 0.007
	Class 2	0.23	<0.001	$Cov_{(linear,\ intercept)}$ = -0.013
	Class 3	-0.16	<0.001	
	Class 4	-0.06	0.069	
Quadratic	Class 1	0.01	0.128	$\sigma^2_{quadratic\ slope}$ = 0.001
	Class 2	-0.12	<0.001	$Cov_{(quadratic,\ intercept)}$ = -0.010
	Class 3	-0.01	0.087	$Cov_{(quadratic,\ linear)}$ = -0.0003
	Class 4	-0.01	0.101	
Cubic	Class 1	-0.002	0.050	$\sigma^2_{cubic\ slope}$ = 10^{-5}
	Class 2	0.01	<0.001	$Cov_{(cubic,\ intercept)}$ = 0.001
	Class 3	0.003	<0.001	$Cov_{(cubic,\ linear)}$ = -0.00001
	Class 4	0.004	<0.001	$Cov_{(cubic,\ quadratic)}$ = -0.0001
				RSE=0.007

RSE, residual standard error.

© 2026 Deng R et al. *JAMA Network Open*.

eTable 7. Latent Class Growth Mixture Models Results of Model Fitting Process in Boys in LSAC

No. of class	Polynomial degree	BIC	Entropy	% Participants per class	AvePP	Proportion of AvePP > 70%
	Linear	29247.35	1.00	100	NA	NA
1	Quadratic	28186.49	1.00	100	NA	NA
	Cubic	24486.47	1.00	100	NA	NA
	Linear	26614.63	0.00	53.11/46.89	0.51/0.49	NA
2	Quadratic	25550.74	0.63	77.54/22.46	0.91/0.82	91.62/74.68
	Cubic	24408.76	0.80	9.23/90.77	0.81/0.96	74.84/96.29
	Linear	26571.59	0.67	0.81/81.54/17.64	0.65/0.88/0.82	50.00/90.25/74.34
3	Quadratic	25569.83	0.60	69.24/23.68/7.08	0.82/0.85/0.69	82.06/78.43/42.62
	Cubic	24394.79	0.76	5.75/10.04/84.21	0.77/0.72/0.93	62.63/55.49/91.87
	Linear	26592.74	0.48	52.7/30.99/1.39/14.92	0.69/0.59/0.64/0.78	50.11/0.94/33.33/65.37
4	Quadratic	25537.38	0.69	1.39/75.68/8.13/14.8	0.68/0.88/0.74/0.74	50.00/86.12/57.14/56.86
	Cubic	24411.01	0.68	25.54/7.2/10.1/57.17	0.73/0.78/0.69/0.72	52.95/63.71/46.55/55.94
	Linear	26615.09	0.35	0/37.78/3.25/44.05/14.92	0/0.41/0.51/0.54/0.78	0/0/14.29/0.66/64.98
5	Quadratic	25558.45	0.60	5.05/8.36/66.51/7.89/12.19	0.71/0.51/0.80/0.71/0.66	49.43/4.86/78.18/55.15/44.76
	**Cubic**	**24398.81**	**0.69**	**5.17/9.92/61.11/20.2/3.6**	**0.77/0.77/0.83/0.74/0.73**	**69.66/64.91/81.48/59.48/51.61**

No. class, latent class number of the model; BIC, the Bayesian information Criterion; AvePP, Average posterior probabilities; NA, not applicable.

© 2026 Deng R et al. *JAMA Network Open*.

eTable 8. Parameter Estimates for the Best-Fitting 4-Class Cubic Latent Class Growth Mixture Model of Boys in LSAC

Parameters	Class	Fixed effect		Random effect: variance-covariance matrix
		Estimated value	*P*	
Intercept	Class 1	1.73	<0.001	$\sigma^2_{intercept}$ = 0.522
	Class 2	-0.27	0.023	
	Class 3	0.61	<0.001	
	Class 4	0.95	<0.001	
	Class 5	2.72	<0.001	
Linear	Class 1	0.40	<0.001	$\sigma^2_{linear\ slope}$ = 0.007
	Class 2	-0.23	<0.001	Cov $_{(linear,\ intercept)}$ = -0.03
	Class 3	-0.16	<0.001	
	Class 4	0.02	0.364	
	Class 5	0.04	0.414	
Quadratic	Class 1	-0.01	0.001	$\sigma^2_{quadratic\ slope}$ = 0.0003
	Class 2	-0.01	<0.001	Cov $_{(quadratic,\ intercept)}$ = 0.001
	Class 3	-0.01	<0.001	Cov $_{(quadratic,\ linear)}$ = -0.01
	Class 4	0.003	0.331	
	Class 5	-0.05	<0.001	
Cubic	Class 1	-0.006	<0.001	$\sigma^2_{cubic\ slope}$ = 2×10^{-5}
	Class 2	0.005	<0.001	Cov $_{(cubic,\ intercept)}$ = 0.002
	Class 3	0.006	<0.001	Cov $_{(cubic,\ linear)}$ = -0.0002
	Class 4	0.004	<0.001	Cov $_{(cubic,\ quadratic)}$ = -0.0001
	Class 5	0.006	<0.001	RSE=0.005

RSE, residual standard error.

© 2026 Deng R et al. *JAMA Network Open*.

eTable 9. Latent Class Growth Mixture Models Results of Model Fitting Process in Boys in TBCS

No. of class	Polynomial degree	BIC	Entropy	% Participants per class	AvePP	Proportion of AvePP > 70%
	Linear	17368.31	1.00	100	NA	NA
1	Quadratic	17163.42	1.00	100	NA	NA
	Cubic	17013.16	1.00	100	NA	NA
	Linear	17381.24	0.42	57.01/42.99	0.83/0.80	77.99/69.53
2	Quadratic	17151.28	0.63	38.93/61.07	0.85/0.91	79.15/90.03
	Cubic	17019.37	0.60	58.12/41.88	0.90/0.85	88.57/83.26
	Linear	17400.13	0.28	47.42/0/52.58	0.47/0/0.73	0/0/56.84
3	Quadratic	17111.48	0.73	4.98/55.35/39.67	0.83/0.89/0.86	74.07/88.67/81.40
	Cubic	16992.2	0.73	54.24/4.43/41.33	0.88/0.83/0.87	86.05/79.17/84.82
	Linear	17400.82	0.68	36.35/51.11/0.37/12.18	0.87/0.78/0.98/0.77	84.77/77.98/100/74.24
4	Quadratic	17128.44	0.63	38.75/13.47/4.8/42.99	0.86/0.68/0.84/0.75	80.95/43.84/76.92/66.52
	**Cubic**	**16998.16**	**0.76**	**2.21/40.96/15.68/41.14**	**0.84/0.83/0.86/0.90**	**83.33/81.98/82.35/89.24**
	Linear	17417.61	0.58	38.93/40.77/10.7/9.41/0.18	0.64/0.84/0.58/0.66/1.00	35.07/78.28/18.97/37.25/100
5	Quadratic	17142.49	0.63	9.96/32.66/17.71/36.72/2.95	0.67/0.77/0.84/0.73/0.81	40.74/66.10/79.17/58.79/62.50
	Cubic	17024.52	0.70	3.69/4.61/16.42/41.14/34.13	0.86/0.61/0.83/0.87/0.75	90/24/76.40/85.65/63.24

No. class, latent class number of the model; BIC, the Bayesian information Criterion; AvePP, Average posterior probabilities; NA, not applicable.

© 2026 Deng R et al. *JAMA Network Open*.

eTable 10. Parameter Estimates for the Best-Fitting 4-Class Cubic Latent Class Growth Mixture Model of Boys in TBCS

Parameters	Class	Fixed effect		Random effect: variance-covariance matrix
		Estimated value	*P*	
Intercept	Class 1	2.99	<0.001	σ^2_{int} = 0.654
	Class 2	0.19	0.017	
	Class 3	-0.29	0.016	
	Class 4	0.70	<0.001	
Linear	Class 1	0.73	<0.001	$\sigma^2_{linear\ slope}$ = 0.015
	Class 2	-0.10	<0.001	Cov $_{(linear,\ intercept)}$ = 0.013
	Class 3	-0.21	<0.001	
	Class 4	0.19	<0.001	
Quadratic	Class 1	-0.04	0.147	$\sigma^2_{quadratic\ slope}$ = 0.001
	Class 2	-0.004	0.398	Cov $_{(quadratic,\ intercept)}$ = -0.019
	Class 3	-0.03	<0.001	Cov $_{(quadratic,\ linear)}$ = -0.001
	Class 4	0.03	<0.001	
Cubic	Class 1	-0.006	0.038	$\sigma^2_{cubic\ slope}$ = 10^{-5}
	Class 2	0.004	<0.001	Cov $_{(cubic,\ intercept)}$ = 0.001
	Class 3	0.006	<0.001	Cov $_{(cubic,\ linear)}$ = -0.001
	Class 4	-0.004	<0.001	Cov $_{(cubic,\ quadratic)}$ = -0.0001
				RSE=0.008

RSE, residual standard error.

© 2026 Deng R et al. *JAMA Network Open*.

eFigure 2. Examples of Calculation of the Cumulative Exposures to BMI *z* Scores Within (1,2] (Filled With Blue) and Cumulative Exposures to BMI *z* Scores >2 (Filled With Yellow) Derived From the BMI *z* Score Trajectories of 3 Random Children

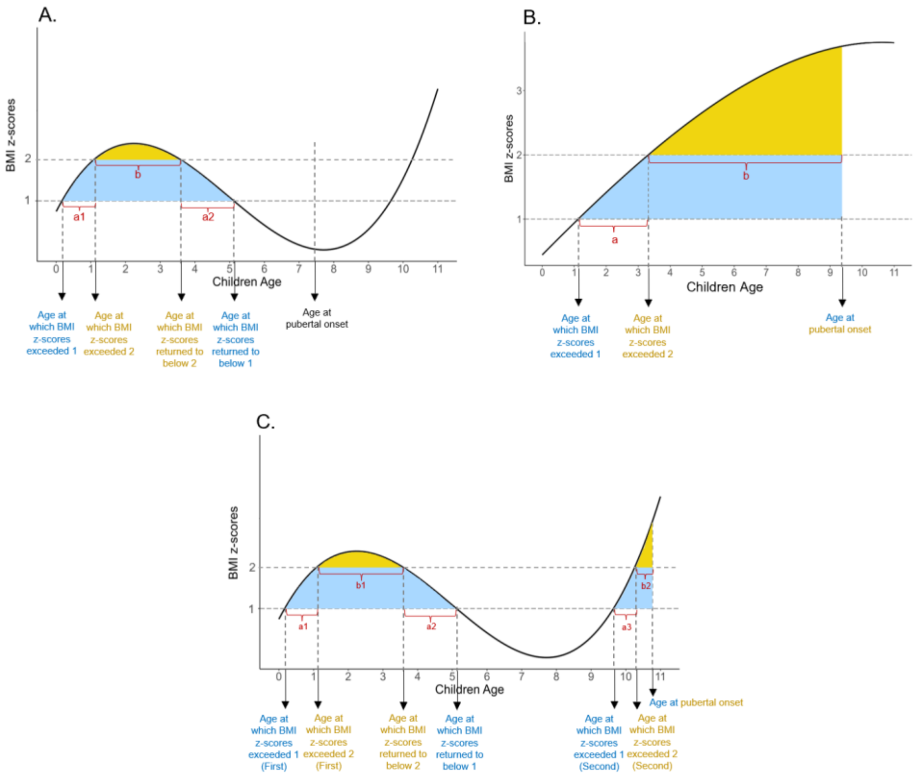

Note: The cut-off ages of cumulative exposures to BMI z scores within (1,2] were indicated with arrows and highlighted in blue font, and those of cumulative exposures to BMI z scores >2 were highlighted in yellow font.

a/a1/a2/a3, duration of BMI z scores within (1,2]. b/b1/b2, duration of BMI z scores >2.

© 2026 Deng R et al. *JAMA Network Open*.

For child C, the duration of BMI z scores within (1,2] was defined as the sum of a1, a2 and a3, and the duration of BMI z scores >2 was defined as the sum of b1 and b2. Accordingly, the cumulative exposures to BMI z scores within (1,2] and the cumulative exposures to BMI z scores >2 were defined as the sum of all the blue parts and yellow parts, respectively.

The age at pubertal onset here was assigned on an individual basis and as follows: the age at the first round for children who had already entered puberty at baseline (which was also the age at first reported puberal onset), the age at first reported pubertal onset for children with new-onset puberty during follow-up, and age at the last round for those who had not entered puberty by the end of the study.

© 2026 Deng R et al. *JAMA Network Open*.

eTable 11. Tests of Proportional Hazards Assumption for Each Exposure Variable

	LSAC		TBCS	
	Girls	Boys	Girls	Boys
	P value	*P* value	*P* value	*P* value
Cumulative exposures to BMI z scores within (1,2]	0.867	**0.010**	0.815	0.126
Duration of BMI z scores within (1,2]	0.648	0.168	0.846	0.334
Average cumulative exposures to BMI z scores within (1,2]	0.474	0.226	0.646	0.921
Cumulative exposures to BMI z scores >2	0.641	**0.052**	0.700	0.777
Duration of BMI z scores >2	0.939	**0.030**	0.975	0.055
Average cumulative exposures to BMI z scores > 2	0.699	**0.029**	0.494	0.734
Cumulative exposure to BMI z scores within (1,2]				
During the age period of 3 to 4 years	0.296	**0.022**	0.412	0.762
Outside the age period of 3 to 4 years	0.958	**0.016**	0.860	0.106
Cumulative exposure to BMI z scores > 2				
During the age period of 3 to 4 years	0.495	0.488	0.389	0.159
Outside the age period of 3 to 4 years	0.706	**0.040**	0.769	0.904

Models were adjusted for maternal education level, delivery mode, breastfeeding, fruit intake frequency, vegetable intake frequency in both cohorts, parity and children's choice to spend free time in LSAC, maternal age at delivery and weekly frequency of vigorous exercise in TBCS, respectively.

P values for the adjusted covariates in each model were not shown due to space constraints; all satisfied the proportional hazards assumption.

Variables in bold violated the proportional hazards assumption.

© 2026 Deng R et al. *JAMA Network Open*.

eTable 12. Characteristics of Participants by Sex

	Girls	Boys	t/χ^2	*p* value
LSAC (N =3,354)				
n (%)	1631 (48.63)	1723 (51.37)		
Age at the last round	14.83±0.69	14.83±0.53	0.566	0.571
Proportion of pubertal onset by the last round	1609 (98.65)	1575 (91.41)	93.717	<0.001
Ethnicity			0.032	0.858
Australia born	1625 (99.63)	1716 (99.59)		
Others	6 (0.37)	7 (0.41)		
Parity			2.924	0.232
Singleton	1575 (96.57)	1664 (96.58)		
Polyembryony	56 (3.43)	59 (3.42)		
Delivery mode			15.258	<0.001
Spontaneous delivery	1044 (66.24)	1000 (60.17)		
Cesarean section	435 (27.60)	517 (31.11)		
Others	97 (6.15)	145 (8.72)		
Maternal education level			2.374	0.882
Year 12 or equivalent	242 (20.74)	272 (21.42)		
Year 11 or equivalent	376 (32.22)	401 (31.57)		
Year 10 or equivalent	168 (14.40)	177 (13.94)		
Year 9 or below	381 (32.65)	420 (33.07)		
Breastfeeding at three months			0.617	0.432
No	408 (25.08)	452 (26.26)		
Yes	1219 (74.92)	1269 (73.74)		
TBCS (N =1,105)				

© 2026 Deng R et al. *JAMA Network Open*.

n (%)	563 (50.95)	542 (49.05)		
Age at the last round	10.62±0.60	10.65±0.61	0.709	0.480
Proportion of pubertal onset by the last round (%)	456 (80.99)	319 (58.86)	64.618	<0.001
Ethnicity			0.652	0.419
Han	534 (97.62)	517 (96.82)		
Others	13 (2.38)	17 (3.18)		
Maternal age at delivery	27.86 (4.55)	27.80 (4.23)	-0.236	0.814
Delivery mode			0.560	0.454
Spontaneous delivery	152 (27.00)	157 (29.02)		
Cesarean section	411 (73.00)	384 (70.98)		
Maternal education level			0.626	0.731
Junior high school and below	101 (17.94)	102 (18.85)		
High school or equivalent	260 (46.18)	257 (47.50)		
Bachelor degree and above	202 (35.88)	182 (33.64)		
Feeding method			0.708	0.702
Breast feeding	314 (57.40)	301 (56.37)		
Artificial feeding	82 (14.99)	90 (16.85)		
Mixed feeding	151 (27.61)	143 (26.78)		

Differences were tested by student's t-test for continuous variables and χ^2 test for categorical variables.

© 2026 Deng R et al. *JAMA Network Open*.

eTable 13. Sensitivity Analyses of Childhood BMI *z* Score Trajectory Groups Associated With Risk of Pubertal Onset Based on a Discrete-Time Survival Model, With Comparison With Primary Survival Analyses

	Girls		Boys	
	Model 1 [a]	Model 2 [b]	Model 1	Model 2
	HR (95% *CI*)	*HR* (95% *CI*)	*HR* (95% *CI*)	*HR* (95% *CI*)
LSAC				
High-level	2.80 (1.69, 4.63) ***	2.88 (1.85, 4.49) ***	1.28 (0.97, 1.70)	1.31 (0.98, 1.75)
Early-increasing	1.93 (1.44, 2.59) ***	2.30 (1.61, 3.26) ***	1.19 (0.94, 1.52)	1.15 (0.80, 1.64)
Medium-increasing	1.58 (1.41, 1.78) ***	1.64 (1.43, 1.88) ***	1.16 (1.01, 1.32) *	1.15 (1.01, 1.32) *
Stable	1.00 (ref)	1.00 (ref)	1.00 (ref)	1.00 (ref)
Low-stable	0.71 (0.60, 0.84) ***	0.72 (0.62, 0.83) ***	0.77 (0.64, 0.92) **	0.72 (0.60, 0.85) ***
TBCS				
High-level	1.33 (0.84, 2.13)	1.33 (0.86, 2.06)	0.61 (0.25, 1.51)	0.60 (0.22, 1.66)
Increasing	1.35 (1.04, 1.74) *	1.36 (1.03, 1.78) *	0.93 (0.73, 1.19)	0.94 (0.74, 1.19)
Stable	1.00 (ref)	1.00 (ref)	1.00 (ref)	1.00 (ref)
Low-stable	0.75 (0.60, 0.93) *	0.76 (0.61, 0.94) *	0.92 (0.66, 1.28)	0.93 (0.66, 1.32)

[a] Model 1: primary survival analyses using Cox proportional model.

[b] Model 2: sensitivity analyses using discrete-time survival model.

Both Model 1 and Model 2 were adjusted for maternal education level, delivery mode, breastfeeding, fruit intake frequency, vegetable intake frequency in both cohorts, parity and children's choice to spend free time in LSAC, maternal age at delivery and weekly frequency of vigorous exercise in TBCS, respectively.

HR, hazard ratio; *CI*, confidence interval; BMI, body mass index; LSAC, longitudinal study of Australian children; TBCS, Tianjin birth cohort study.

* $p < 0.05$, ** $p < 0.01$, *** $p < 0.001$

© 2026 Deng R et al. *JAMA Network Open*.

eFigure 3. Nonlinear Associations Between Cumulative Exposures to Different Levels of Adiposity and Risk of Pubertal Onset

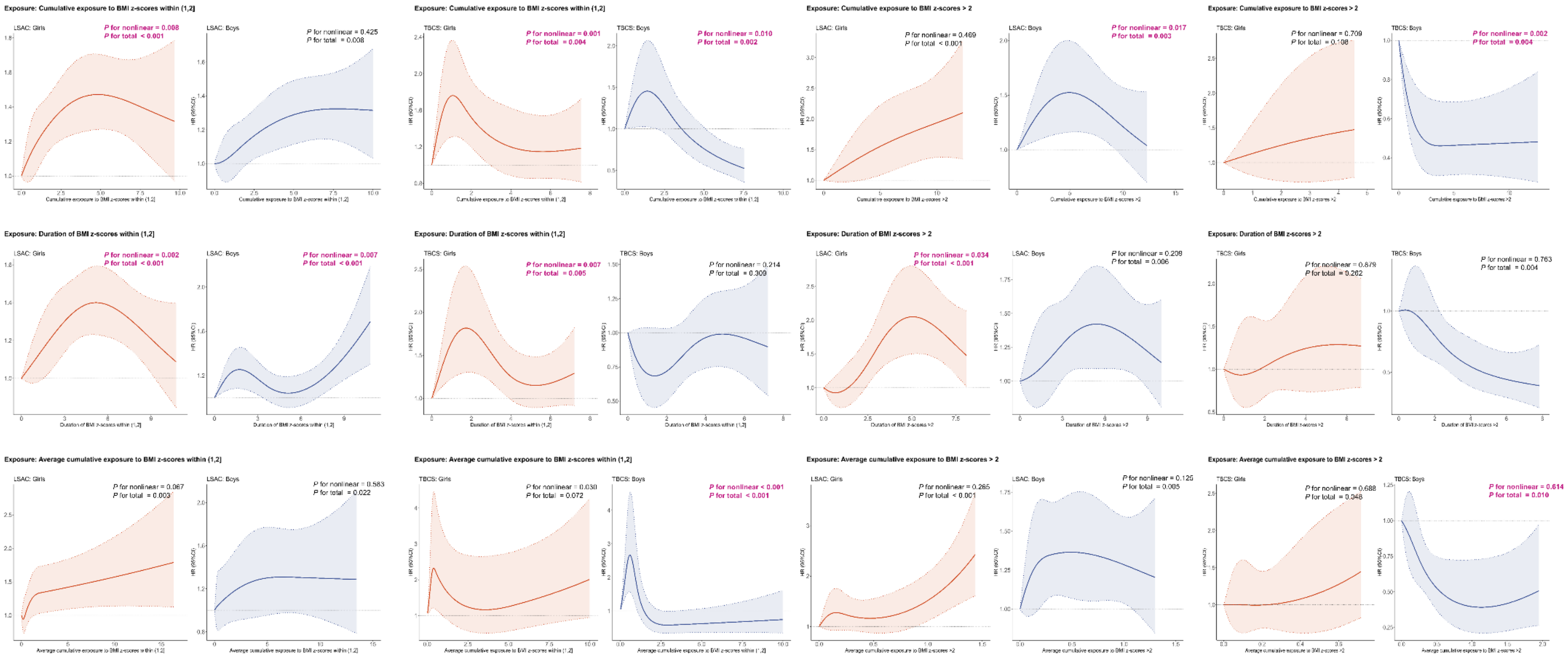

Note: Models were adjusted for maternal education level, delivery mode, breastfeeding, fruit intake frequency, vegetable intake frequency in both cohorts, parity and children's choice to spend free time in LSAC, maternal age at delivery and weekly frequency of vigorous exercise in TBCS, respectively. Purple bold font indicates significant nonlinear associations.

© 2026 Deng R et al. *JAMA Network Open*.

eTable 14. Time-Varying Effects for Exposures Violating the Proportional Hazards Assumption (Only Observed in Boys of LSAC)

	Boys					
	Model 1 [a]			Model 2 [b]		
	Time Interaction *HR* (95%*CI*)	Annual Attenuation Rate	Time (y) when *HR*=1	Time Interaction *HR* (95%*CI*)	Annual Attenuation Rate	Time (y) when *HR*=1
**LSAC**						
Cumulative exposures to BMI z scores within (1,2]	0.98 (0.97, 0.99) **	1.85%	14.67	0.98 (0.97, 0.99) **	2.04%	14.08
Duration of BMI z scores >2	0.98 (0.96, 0.99) *	1.90%	14.16	0.98 (0.96, 0.99) *	2.11%	13.46
Average cumulative exposures to BMI z scores > 2	0.86 (0.75, 0.98) *	14.31%	14.13	0.84 (0.73, 0.98) *	15.60%	13.40
Cumulative exposure to BMI z scores within (1,2]						
During the age period of 3 to 4 years	0.87 (0.77, 0.97) *	13.14%	13.16	0.87 (0.77, 0.98) *	13.26%	12.81
Outside the age period of 3 to 4 years	0.98 (0.97, 0.99) **	1.88%	14.28	0.98 (0.96, 0.99) **	2.10%	13.92
Cumulative exposure to BMI z scores > 2						
Outside the age period of 3 to 4 years	0.98 (0.96, 0.99) *	1.87%	13.60	0.98 (0.96, 0.99) *	2.05%	12.82

[a] Model 1 was null model.

[b] Model 2 were adjusted for maternal education level, delivery mode, breastfeeding, fruit intake frequency, vegetable intake frequency in both cohorts, parity and children's choice to spend free time in LSAC, maternal age at delivery and weekly frequency of vigorous exercise in TBCS, respectively.

Baseline *HR*s are reported in the primary results (Table 2 and Table 3). This table only presents time-varying effects.

HR, hazard ratio; *CI*, confidence interval; BMI, body mass index; LSAC, longitudinal study of Australian children

* $p < 0.05$, ** $p < 0.01$, *** $p < 0.001$

© 2026 Deng R et al. *JAMA Network Open*.

eTable 15. Sensitivity Analyses of Association of Cumulative Exposures to Different Levels of Adiposity With Risk of Pubertal Onset Based on Discrete-Time Survival Model, With Comparison With Primary Survival Analyses

	Girls		Boys	
	**Model 1** [a]	**Model 2** [b]	**Model 1**	**Model 2**
	***HR* (95% *CI*)**	***HR* (95% *CI*)**	***HR* (95% *CI*)**	***HR* (95% *CI*)**
LSAC				
Cumulative exposures to BMI z scores within (1,2]	1.27 (1.15, 1.42) [***c]	1.05 (1.03, 1.08) [***]	1.33 (1.12, 1.58) [***d]	1.04 (1.02, 1.07) [**]
Duration of BMI z scores within (1,2]	1.27 (1.15, 1.42) [*** c]	1.04 (1.02, 1.05) [***]	1.16 (1.04, 1.29) [** c]	1.03 (1.01, 1.05) [**]
Average cumulative exposures to BMI z scores within (1,2]	1.04 (1.01, 1.06) [**]	1.03 (1.00, 1.06) [*]	1.02 (0.99, 1.05)	1.03 (0.99, 1.05)
Cumulative exposures to BMI z scores >2	1.06 (1.03, 1.10) [***]	1.06 (1.03, 1.10) [***]	1.21 (1.04, 1.41) [* c]	1.02 (0.99, 1.06)
Duration of BMI z scores >2	1.31 (1.09, 1.58) [** c]	1.08 (1.04, 1.12) [***]	1.33 (1.07, 1.65) [* d]	1.03 (1.00, 1.07) [*]
Average cumulative exposures to BMI z scores >2	1.68 (1.29, 2.19) [***]	1.75 (1.30, 2.36) [***]	9.69 (1.66, 56.57) [* d]	1.30 (0.97, 1.73)
TBCS				
Cumulative exposures to BMI z scores within (1,2]	1.27 (1.05, 1.55) [* c]	1.03 (0.99, 1.08)	0.73 (0.58, 0.92) [** c]	0.93 (0.89, 0.97) [**]
Duration of BMI z scores within (1,2]	1.27 (1.05, 1.55) [* c]	1.04 (1.00, 1.08) [*]	0.99 (0.94, 1.04)	0.99 (0.94, 1.05)
Average cumulative exposures to BMI z scores within (1,2]	1.02 (0.96, 1.09)	1.03 (0.97, 1.08)	0.74 (0.59, 0.93) [** c]	0.90 (0.81, 0.99) [*]
Cumulative exposures to BMI z scores >2	1.03 (1.02, 1.04) [***]	1.07 (0.99, 1.14)	0.95 (0.59, 0.95) [*c]	0.94 (0.88, 1.01)
Duration of BMI z scores >2	1.03 (0.96, 1.09)	1.03 (0.97, 1.08)	0.88 (0.82, 0.94) [***]	0.88 (0.82, 0.93) [***]
Average cumulative exposures to BMI z scores >2	1.18 (1.07, 1.29) [***]	1.91 (1.09, 3.36) [*]	0.75 (0.59, 0.95) [* c]	0.59 (0.35, 1.01)

[a] Model 1: primary survival analyses using Cox proportional model.

[b] Model 2: sensitivity analyses using discrete-time survival model.

Both Model 1 and Model 2 were adjusted for maternal education level, delivery mode, breastfeeding, fruit intake frequency, vegetable intake frequency in both cohorts, parity and children's choice to spend free time in LSAC, maternal age at delivery and weekly frequency of vigorous exercise in TBCS, respectively.

[c] As noted in the main tables of primary survival analyses, this variable exhibited a nonlinear association rather than a linear association. The nonlinear curves and corresponding P-values were presented in eFigure3.The effect size here indicated the zero-indicator-effect.

[d] As noted in the main tables of primary survival analyses, this referred to the baseline *HR* (95% CI) for variables violating the proportional hazards assumption.

© 2026 Deng R et al. *JAMA Network Open*.

HR, hazard ratio; *CI*, confidence interval; BMI, body mass index; LSAC, longitudinal study of Australian children; TBCS, Tianjin birth cohort study.

* $p < 0.05$, ** $p < 0.01$, *** $p < 0.001$

© 2026 Deng R et al. *JAMA Network Open*.

eTable 16. Association of Overall BMI Status With Age and Risk of Pubertal Onset

	Girls		Boys	
	**Model 1** [a]	**Model 2** [b]	**Model 1**	**Model 2**
	β/HR **(95%** ***CI*****)** [c]	***β/HR*** **(95%** ***CI*****)**	***β/HR*** **(95%** ***CI*****)**	***β/HR*** **(95%** ***CI*****)**
Age at pubertal onset				
LSAC				
Maintaining normal weight	0.00 (ref)	0.00 (ref)	0.00 (ref)	0.00 (ref)
BMI z scores ever within (1,2]	-0.44 (-0.61, -0.26) ***	-0.40 (-0.58, -0.23) ***	-0.22 (-0.40, -0.03) *	-0.20 (-0.39, -0.01) *
BMI z scores ever >2	-0.56 (-0.89, -0.23) **	-0.52 (-0.85, -0.19) **	-0.62 (-0.88, -0.36) ***	-0.61 (-0.88, -0.35) ***
TBCS				
Maintaining normal weight	0.00 (ref)	0.00 (ref)	0.00 (ref)	0.00 (ref)
BMI z scores ever within (1,2]	-0.38 (-0.63, -0.13) ***	-0.36 (-0.62, -0.11) ***	0.22 (-0.21, 0.65)	0.23 (-0.20, 0.66)
BMI z scores ever >2	-0.22 (-0.57, 0.13)	-0.22 (-0.58, 0.13)	0.51 (0.15, 0.86) ***	0.54 (0.18, 0.90) ***
Risk of pubertal onset by the last round				
LSAC				
Maintaining normal weight	1.00 (ref)	1.00 (ref)	1.00 (ref)	1.00 (ref)
BMI z scores ever within (1,2]	1.27 (1.14, 1.41) ***	1.24 (1.12, 1.38) ***	1.12 (1.01, 1.25) *	1.12 (0.99, 1.25)
BMI z scores ever >2	1.42 (1.19, 1.71) ***	1.40 (1.16, 1.68) ***	1.32 (1.14, 1.53) ***	1.32 (1.14, 1.54) ***

© 2026 Deng R et al. *JAMA Network Open*.

TBCS				
Maintaining normal weight	1.00 (ref)	1.00 (ref)	1.00 (ref)	1.00 (ref)
BMI z scores ever within (1,2]	1.28 (1.04, 1.58) *	1.27 (1.02, 1.58) *	0.82 (0.61, 1.10)	0.82 (0.60, 1.10)
BMI z scores ever >2	1.20 (0.89, 1.61)	1.20 (0.89, 1.62)	0.73 (0.57, 0.94) *	0.71 (0.55, 0.92) **

[a] Model 1 was null model.

[b] Model 2 were adjusted for maternal education level, delivery mode, breastfeeding, fruit intake frequency, vegetable intake frequency in both cohorts, parity and children's choice to spend free time in LSAC, maternal age at delivery and weekly frequency of vigorous exercise in TBCS, respectively.

[c] This referred to *β* (95%*CI*) when the outcome was age at pubertal onset, and *HR* (95%*CI*) when the outcome was risk of pubertal onset by the last round.

HR, hazard ratio; *CI*, confidence interval; BMI, body mass index; LSAC, longitudinal study of Australian children; TBCS, Tianjin birth cohort study.

* $p < 0.05$, ** $p < 0.01$, *** $p < 0.001$

© 2026 Deng R et al. *JAMA Network Open*.

eTable 17. Association of Cumulative Exposures to Different Levels of Adiposity With Age and Risk of Pubertal Onset Stratified by Overall BMI Status

	Girls		Boys	
	**Model 1 [a]**	**Model 2 [b]**	**Model 1**	**Model 2**
	***β/HR* (95% *CI*) [c]**	***β/HR* (95% *CI*)**	***β/HR* (95% *CI*)**	***β/HR* (95% *CI*)**
Age at pubertal onset				
LSAC				
BMI z scores ever within (1,2]				
Cumulative exposures to BMI z scores within (1,2]	-0.08 (-0.16, -0.01) *	-0.08 (-0.16, -0.01) *	-0.12 (-0.21, -0.02) *	-0.11 (-0.20, -0.01) *
Duration of BMI z scores within (1,2]	-0.05 (-0.09, -0.002) *	-0.04 (-0.09, 0.009)	-0.07 (-0.12, -0.02) **	-0.07 (-0.12, -0.02) *
Average cumulative exposures to BMI z scores within (1,2]	-0.98 (-1.68, -0.28) **	-0.98 (-1.68, -0.27) **	-0.79 (-1.64, 0.06)	-0.74 (-1.58, 0.10)
BMI z scores ever above 2				
Cumulative exposures to BMI z scores >2	-0.08 (-0.16, 0.004)	-0.05 (-0.13, 0.04)	-0.06 (-0.12, -0.002) *	-0.05 (-0.11, 0.02)
Duration of BMI z scores >2	-0.11 (-0.21, 0.002)	-0.08 (-0.19, 0.03)	-0.08 (-0.16, -0.01) *	-0.06 (-0.14, 0.01)
Average cumulative exposures to BMI z scores >2	-0.78 (-1.49, -0.06) *	-0.52 (-1.25, 0.21)	-0.63 (-1.19, -0.08) *	-0.48 (-1.06, 0.10)
TBCS				
BMI z scores ever within (1,2]				

© 2026 Deng R et al. *JAMA Network Open*.

Cumulative exposures to BMI z scores within (1,2]	0.06 (-0.15, 0.26)	0.03 (-0.18, 0.24)	-0.68 (-1.14, -0.21) [**]	-0.64 (-1.12, -0.16) [**]
Duration of BMI z scores within (1,2]	0.01 (-0.10, 0.13)	0.007 (-0.11, 0.12)	-0.34 (-0.56, -0.11) [**]	-0.32 (-0.54, -0.10) [**]
Average cumulative exposures to BMI z scores within (1,2]	-0.40 (-1.81, 1.02)	-0.59 (-2.02, 0.83)	-4.06 (-6.30, -1.83) [***]	-4.14 (-6.53, -1.75) [**]
BMI z scores ever >2				
Cumulative exposures to BMI z scores >2	-0.10 (-0.20, 0.005)	-0.11 (-0.21, -0.002) [*]	0.02 (-0.04, 0.07)	0.03 (-0.03, 0.09)
Duration of BMI z scores >2	-0.04 (-0.15, 0.07)	-0.05 (-0.17, 0.08)	0.19 (0.08, 0.31) [**]	0.21 (0.10, 0.32) [**]
Average cumulative exposures to BMI z scores >2	-1.06 (-1.86, -0.25) [*]	-1.22 (-2.08, -0.36) [**]	0.10 (-0.36, 0.55)	0.23 (-0.24, 0.70)
Risk of pubertal onset by the last round				
LSAC				
BMI z scores ever within (1,2]				
Cumulative exposures to BMI z scores within (1,2]	1.05 (1.01, 1.10) [*]	1.05 (1.01, 1.10) [*]	1.04 (0.98, 1.10)	1.04 (0.98, 1.10)
Duration of BMI z scores within (1,2]	1.03 (1.00, 1.05) [*]	1.03 (1.00, 1.05) [*]	1.02 (0.99, 1.06)	1.03 (0.99, 1.05)
Average cumulative exposures to BMI z scores within (1,2]	1.71 (1.2, 2.61) [*]	1.77 (1.16, 2.71) [**]	1.18 (0.71, 1.97)	1.18 (0.70, 1.99)
BMI z scores ever >2				
Cumulative exposures to BMI z scores >2	1.06 (1.02, 1.10) [**]	1.04 (0.99, 1.08)	1.02 (0.99, 1.04)	1.01 (0.98, 1.04)

© 2026 Deng R et al. *JAMA Network Open*.

Duration of BMI z scores >2	1.08 (1.02, 1.14) *	1.05 (0.99, 1.12)	1.02 (0.99, 1.06)	1.01 (0.97, 1.05)
Average cumulative exposures to BMI z scores > 2	1.70 (1.18, 2.46) **	1.41 (0.93, 2.13)	1.21 (0.92, 1.59)	1.13 (0.85, 1.50)
TBCS				
BMI z scores ever within (1,2]				
Cumulative exposures to BMI z scores within (1,2]	1.00 (0.86, 1.16)	1.03 (0.88, 1.20)	1.43 (1.12, 1.82) **	1.40 (1.08, 1.81) *
Duration of BMI z scores within (1,2]	1.01 (0.93, 1.09)	1.02 (0.93, 1.11)	1.19 (1.06, 1.34) **	1.20 (1.05, 1.36) **
Average cumulative exposures to BMI z scores within (1,2]	1.74 (0.63, 4.78)	2.25 (0.80, 6.36)	13.14 (3.01, 57.33) **	13.85 (2.59, 74.03) **
BMI z scores ever >2				
Cumulative exposures to BMI z scores >2	1.07 (0.98, 1.17)	1.07 (0.98, 1.18)	0.97 (0.92, 1.02)	0.96 (0.90, 1.01)
Duration of BMI z scores > 2	1.02 (0.93, 1.12)	1.02 (0.92, 1.14)	0.83 (0.75, 0.92) **	0.81 (0.73, 0.90) ***
Average cumulative exposures to BMI z scores >2	2.41 (1.14, 5.12) *	2.58 (1.17, 5.71) *	0.78 (0.49, 1.24)	0.68 (0.42, 1.11)

[a] Model 1 was null model.

[b] Model 2 were adjusted for maternal education level, delivery mode, breastfeeding, fruit intake frequency, vegetable intake frequency in both cohorts, parity and children's choice to spend free time in LSAC, maternal age at delivery and weekly frequency of vigorous exercise in TBCS, respectively.

[c] This referred to *β* (95%*CI*) when the outcome was age at pubertal onset, and *HR* (95%*CI*) when the outcome was risk of pubertal onset by the last round.

HR, hazard ratio; *CI*, confidence interval; BMI, body mass index; LSAC, longitudinal study of Australian children; TBCS, Tianjin birth cohort study.

* $p<0.05$, ** $p < 0.01$, *** $p < 0.001$

© 2026 Deng R et al. *JAMA Network Open*.

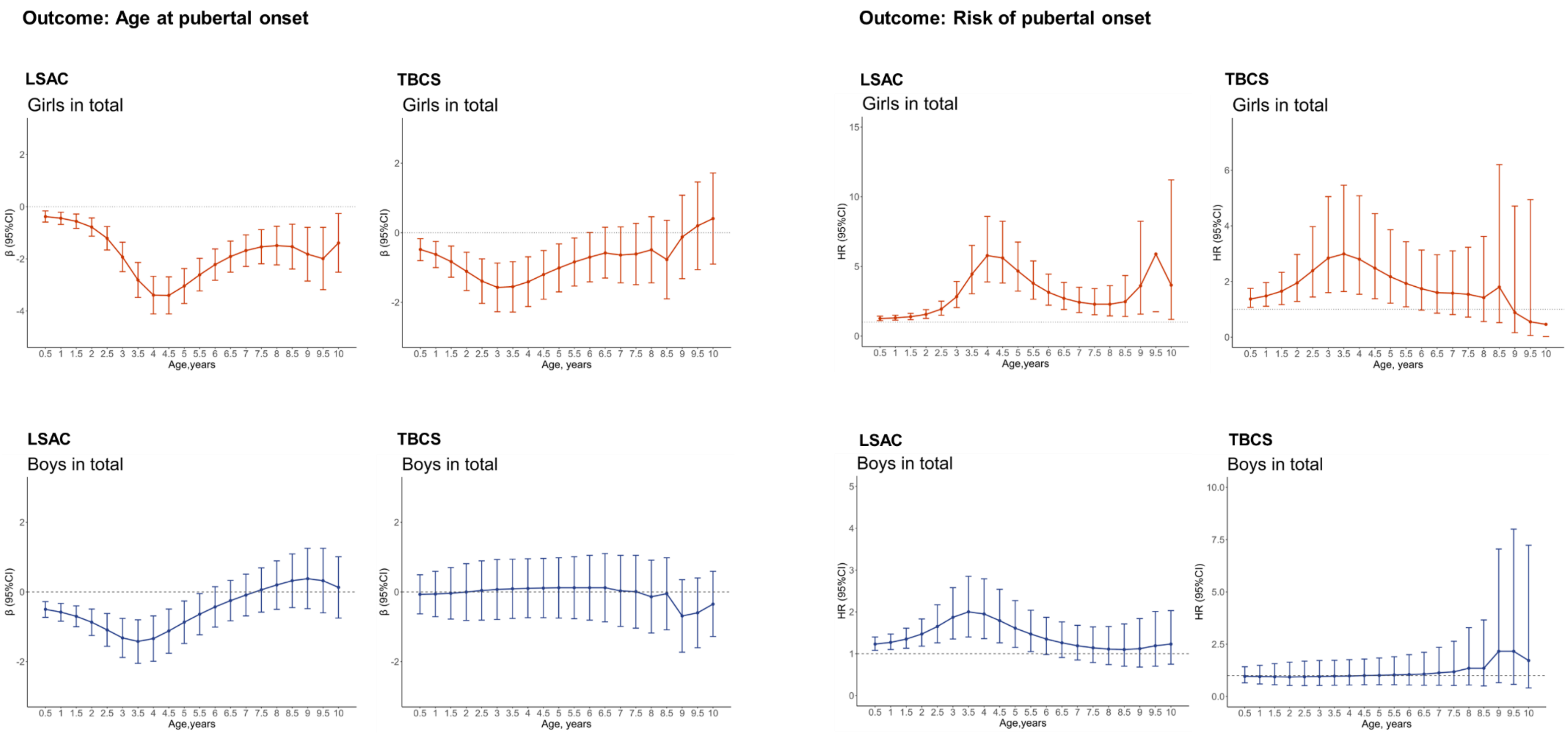

eFigure 4. Associations of Model Estimated Linear Slopes of BMI *z* Scores by Age With Age and Risk of Pubertal Onset Without Adjustment

Note: Some values in both sexes of TBCS were not shown because sample size at these ages were so small that their intervals were too wide to display on the chart.

© 2026 Deng R et al. *JAMA Network Open*.

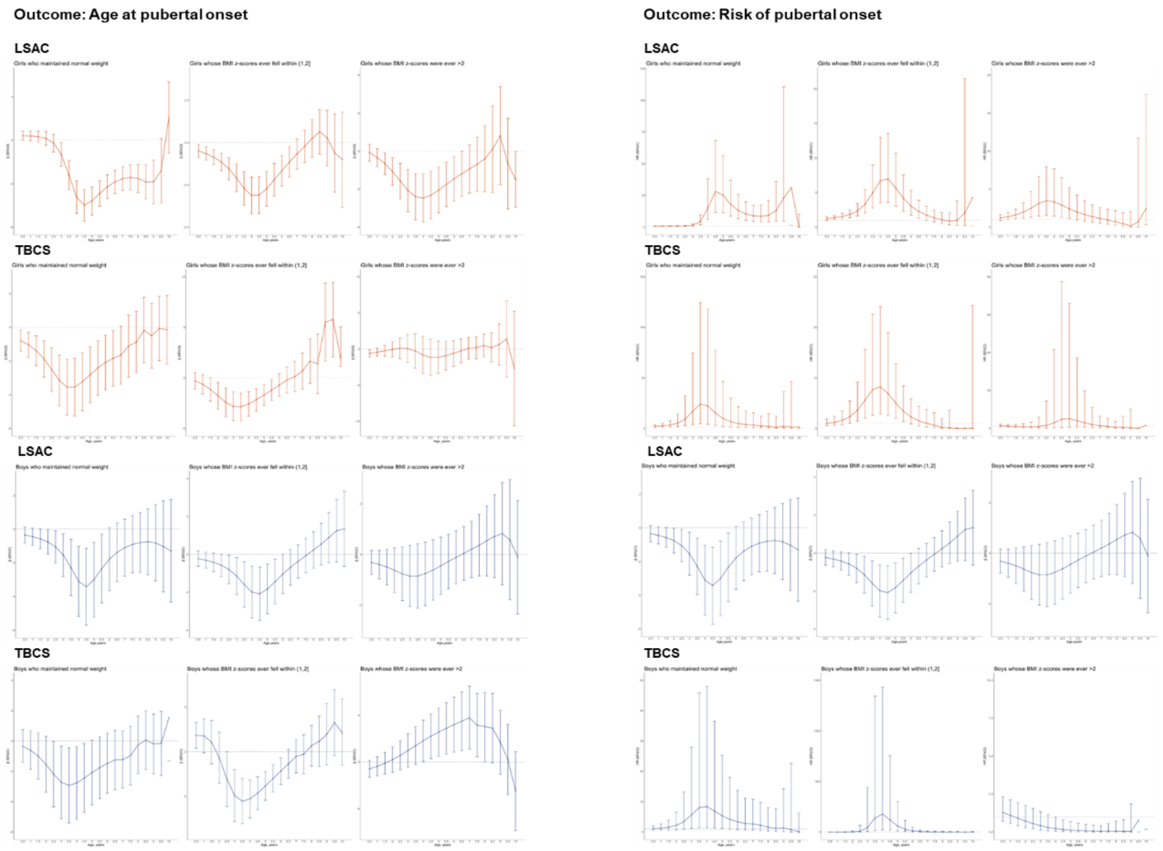

eFigure 5. Associations of Model Estimated Linear Slopes of BMI *z* Scores by Age With Age and Risk of Pubertal Onset Stratified by Total BMI Status

Note: Models were adjusted for maternal education level, delivery mode, breastfeeding, fruit intake frequency, vegetable intake frequency in both cohorts, parity and children's choice to spend free time in LSAC, maternal age at delivery and weekly frequency of vigorous exercise in TBCS, respectively. Some values in both sexes of TBCS were not shown because sample size at these ages were so small that their intervals were too wide to display on the chart.

© 2026 Deng R et al. *JAMA Network Open*.

eTable 18. Sensitivity Analyses of Association of Cumulative Exposure to Different Levels of Adiposity During and Outside of the Sensitive Ages of 3 to 4 Years With Risk of Pubertal Onset Based on Discrete-Time Survival Model, With Comparison With Primary Survival Analyses

	Girls		Boys	
	Model 1 [a]	Model 2 [b]	Model 1	Model 2
	HR (95% *CI*)	*HR* (95% *CI*)	*HR* (95% *CI*)	*HR* (95% *CI*)
LSAC				
Cumulative exposure to BMI z scores within (1,2]				
During the age period	1.30 (1.07, 1.58) **	1.27 (1.02, 1.58) *	6.22 (1.38, 28.00) *d	1.12 (0.91, 1.38)
Outside the age period	1.06 (1.03, 1.08) ***	1.06 (1.03, 1.09) ***	1.34 (1.12, 1.61) **d	1.05 (1.02, 1.07) ***
Cumulative exposure to BMI z scores > 2				
During the age period	1.58 (1.25, 1.99) ***	1.55 (1.27, 1.90) ***	1.24 (0.96, 1.61)	1.29 (1.00, 1.67) *
Outside the age period	1.07 (1.03, 1.11) ***	1.07 (1.03, 1.12) ***	1.31 (1.05, 1.65) * d	1.03 (0.99, 1.07)
TBCS				
Cumulative exposure to BMI z scores within (1,2]				
During the age period	1.26 (0.80, 1.97)	1.23 (0.79, 1.91)	1.29 (0.77, 2.17)	1.29 (0.76, 2.16)
Outside the age period	1.03 (0.99, 1.08)	1.03 (0.99, 1.08)	0.93 (0.87 0.97) **	0.93 (0.89, 0.97) ***
Cumulative exposure to BMI z scores > 2				
During the age period	1.50 (0.97, 2.32)	1.52 (0.98, 2.35)	0.98 (0.57, 1.70)	0.95 (0.46, 1.96)
Outside the age period	1.08 (0.98, 1.17)	1.08 (0.99, 1.16)	0.93 (0.88, 0.99) *	0.93 (0.87, 1.00)

[a] Model 1: primary survival analyses using Cox proportional model.

[b] Model 2: sensitivity analyses using discrete-time survival model.

Both Model 1 and Model 2 were adjusted for maternal education level, delivery mode, breastfeeding, fruit intake frequency, vegetable intake frequency in both cohorts, parity and children's choice to spend free time in LSAC, maternal age at delivery and weekly frequency of vigorous exercise in TBCS, respectively.

[c] The age period here referred to the identified sensitive age window of 3-4 years.

[d] As noted in the main tables of primary survival analyses, this referred to baseline *HR* (95% CI) for variables violating the proportional hazards assumption.

HR, hazard ratio; *CI*, confidence interval; BMI, body mass index; LSAC, longitudinal study of Australian children; TBCS, Tianjin birth cohort study.

© 2026 Deng R et al. *JAMA Network Open*.

* p <0.05, ** p < 0.01, *** p < 0.001

© 2026 Deng R et al. *JAMA Network Open*.
