# Supplementary material for: Prepubertal Growth Trajectory and Pubertal Onset
Source: JAMA Netw Open. 2026 Jun 9;9(6):e2617435. doi: 10.1001/jamanetworkopen.2026.17435 (PMC13250710; doi:10.1001/jamanetworkopen.2026.17435)
Supplement: Supplement 2. — Data Sharing Statement [file jamanetwopen-e2617435-s002.pdf]

Data Sharing Statement

Deng. Prepubertal Growth Trajectory and Pubertal Onset. *JAMA Netw Open*. Published June 09, 2026. doi:10.1001/jamanetworkopen.2026.17435

Data
Data available: No
